# Region-Resolved Quantitative Proteome Profiling Reveals Molecular Dynamics Associated With Chronic Pain in the PNS and Spinal Cord

**DOI:** 10.3389/fnmol.2018.00259

**Published:** 2018-08-14

**Authors:** Allison M. Barry, Julia R. Sondermann, Jan-Hendrik Sondermann, David Gomez-Varela, Manuela Schmidt

**Affiliations:** Max-Planck Institute of Experimental Medicine, Somatosensory Signaling and Systems Biology Group, Goettingen, Germany

**Keywords:** chronic pain, DIA-MS, proteomics, systems biology, neuropathic pain, dorsal root ganglia, sciatic nerve, spinal cord

## Abstract

To obtain a thorough understanding of chronic pain, large-scale molecular mapping of the pain axis at the protein level is necessary, but has not yet been achieved. We applied quantitative proteome profiling to build a comprehensive protein compendium of three regions of the pain neuraxis in mice: the sciatic nerve (SN), the dorsal root ganglia (DRG), and the spinal cord (SC). Furthermore, extensive bioinformatics analysis enabled us to reveal unique protein subsets which are specifically enriched in the peripheral nervous system (PNS) and SC. The immense value of these datasets for the scientific community is highlighted by validation experiments, where we monitored protein network dynamics during neuropathic pain. Here, we resolved profound region-specific differences and distinct changes of PNS-enriched proteins under pathological conditions. Overall, we provide a unique and validated systems biology proteome resource (summarized in our online database painproteome.em.mpg.de), which facilitates mechanistic insights into somatosensory biology and chronic pain—a prerequisite for the identification of novel therapeutic targets.

## Introduction

Chronic pain is a complex maladaptive disease. It has a high incidence and is a major cause of long term disability (Breivik, 2013). Despite prominent scientific advances, our knowledge of the mechanistic underpinnings of chronic pain syndromes remains unsatisfactory (Costigan et al., 2009; Price and Gold, 2017). Effective treatment faces major challenges, largely arising from the fact that therapeutic interventions target widely expressed molecules in multiple tissues, including the central nervous system (Borsook et al., 2014; Price and Gold, 2017). Their modulation is accompanied by severe side effects (Patapoutian et al., 2009; Price and Gold, 2017). Central side effects can be attenuated by selectively targeting the PNS (i.e., afferent fibers, DRG), e.g., by local injections (intranerval, intraganglionic, and epidural). This facilitates effective and safer treatment options (Puljak et al., 2009; Liem et al., 2013; Krames, 2014; Berta et al., 2017b). Interestingly, several studies have demonstrated a crucial role of primary afferent input for the maintenance of chronic pain, e.g., neuropathic pain (Sapunar et al., 2012; Liem et al., 2013; Haroutounian et al., 2014; Krames, 2014), and phantom limb pain (Vaso et al., 2014). While the existence of a peripheral “pain” generator is becoming increasingly accepted, associated changes in molecules and protein networks remain to be fully elucidated (Costigan, 2012; Borsook et al., 2014).

During the past decade, enormous progress has been made toward the identification of molecules implicated in chronic pain. These studies have yielded important insights into genetic variants (Diatchenko et al., 2007, 2013; van Hecke et al., 2015; Zorina-Lichtenwalter et al., 2016; Parisien et al., 2017; Peng et al., 2017) and transcriptome alterations (Grace et al., 2012; Antunes-Martins et al., 2013; Simonetti et al., 2013; Jeong et al., 2016) relevant for chronic pain. Yet, the interpretation of these results is often not straightforward. In particular, genome/transcriptome-based techniques suffer from a limited correspondence between genomic/transcriptomic and proteomic content (Schwanhausser et al., 2011; Sharma et al., 2015; Liu et al., 2016).

As proteins are the functional building blocks of cells, they strongly influence the status-quo of a cell, tissue or even organism. Several proteome studies have thus been conducted on chronic pain (Huang et al., 2008; Olausson et al., 2012, 2015; Sui et al., 2014; Vacca et al., 2014). We are still lacking, however, an integrated systems-level view of protein network organization and dynamics (Alberts, 1998; Rouwette et al., 2015) in the somatosensory system. According to emerging network medicine efforts, the identification of cellular networks and in particular, distinct hubs of disease modules within these networks (Chapman et al., 2008; Barabási et al., 2011; Antunes-Martins et al., 2013; Borsook and Kalso, 2013; Chen et al., 2015), may hold great potential for diagnosis, patient stratification, and design of selective therapeutic interventions for chronic pain conditions (Gereau et al., 2014; Baron et al., 2017; Price and Gold, 2017).

We have previously demonstrated the potential of an emerging mass spectrometry-based proteomic technique (so-called data-independent acquisition mass spectrometry, DIA-MS) to reproducibly profile proteome changes in DRG of two mouse models of chronic pain (Rouwette et al., 2016). Here we considerably extended our approach to build a comprehensive protein compendium (>5000 proteins in total) of three regions at the beginning of the pain neuraxis: the sciatic nerve (SN), the DRG, and the spinal cord (SC). We compared this data to tissue proteomes of the central nervous system which enabled us to define “region-enriched” proteomes. The immense value of these defined region-enriched proteomes becomes apparent by (i) the inclusion of many proteins known to be involved in pain in mice and humans, (ii) the identification of uncharacterized proteins in a tissue-dependent manner, and (iii) the results of our validation experiments resolving profound differences among analyzed tissues and distinct modulations of PNS-enriched proteins during neuropathic pain. Taken together, our findings provide the scientific community with a unique protein-centric systems biology framework for studying the PNS and SC in normal and pathological states. All presented data are summarized in a freely accessible online database at painproteome.em.mpg.de.

## Methods

All experiments were performed by observing standard institutional biosecurity and institutional safety procedures. All workplaces and involved personnel were approved for biosafety level 1 (BSL-1) procedures.

### Mice and behavior

Adult C57Bl/6J male mice (6–8 weeks old at the time of surgery) were bred in-house and kept in a temperature- and humidity-controlled environment under a 12 h light/dark cycle with food and water provided *ad libitum*. All animal experiments were approved and carried out in compliance with institutional guidelines and guidelines of the Landesamt für Verbraucherschutz und Lebensmittelsicherheit of Lower Saxony, Germany (3392-42502-04-13/1077).

The spared nerve injury (SNI) paradigm was induced according to standard protocols as described previously (Decosterd and Woolf, 2000; Minett et al., 2013; Rouwette et al., 2016). Mice were anesthetized with isoflurane (4% induction, 1.8% maintenance) and the SN and its branches, the common peroneal, tibial, and sural nerves, were exposed. Common peroneal and tibial nerves were ligated with a 6.0 silk suture (Braun) and transected distal to the ligation, removing 2–3 mm of each nerve. Care was taken to avoid damaging the sural nerve. Sham surgery was performed in a similar manner without ligating and transecting the nerves. The surgical procedure lasted approximately 15 min. Analgesia during surgery was achieved by Buprenorphine (0.07 mg/kg body weight; Guarnieri et al., 2012). Postsurgical analgesia was achieved by subcutaneous injection of Carprofen (5 mg/kg body weight) for up to 3 days, as necessary. Mechanical sensitivity was assessed 2 days before and 26 days after surgery with a dynamic plantar aesthesiometer (Ugo Basile), as described (Rouwette et al., 2016). Behavioral tests were performed in the light phase in a blinded manner. Mice with neuropathic pain, however, could be easily identified upon the first measurements due to the pronounced mechanical hypersensitivity. All 12 mice which underwent SNI surgery exhibited evident mechanical hypersensitivity (manifested as reduced withdrawal latencies) but were otherwise indistinguishable from Sham surgery mice. None of the 12 mice which underwent Sham surgery exhibited mechanical hypersensitivity. All mice were euthanized with CO_2_ for tissue collection 28 days after surgery.

The following tissues were isolated after Sham surgery or SNI: ipsilateral sciatic nerve, ipsilateral DRG (L3-L5), ipsilateral SC (L3-L5; a reliable and consistent separation of dorsal and ventral horn was technically not feasible), amygdala, anterior cingulate cortex, prefrontal cortex, and cerebellum.

### Protein isolation

Tissues were isolated immediately after CO_2_ euthanization, snap-frozen in liquid nitrogen, and stored at −80°C. Tissue from 4 mice were pooled to obtain 3 replicates per tissue and per condition. Protein isolation was performed as described previously (Bruderer et al., 2017b). In brief, the frozen tissue was homogenized in 4% SDS lysis buffer (4% SDS in 100 mM Tris, 10 mM DTT, 5% glycerol, complete protease inhibitor cocktail (Roche), pH 7.5). Following, the homogenate was incubated at 70°C for 10 min. To remove cell debris, the homogenate was centrifuged at 10,000 × g for 5 min at room temperature. The supernatant corresponds to the whole cell lysates. Subsequently, proteins were precipitated by the addition of 5 × volume pre-chilled acetone (Roth) and incubated for 2 h at −20°C. The protein precipitate was centrifuged at 14,000 × g for 30 min, the pellet washed with ice cold 80% ethanol (AppliedChem), and centrifuged again at 14,000 × g for 30 min. The proteins were air-dried, re-suspended in 2% SDS lysis buffer, and ultimately analyzed by DIA-MS.

To generate the spectral library, i.e., a compendium of all detectable peptides and their physicochemical information necessary to uniquely identify peptides in subsequent DIA-MS experiments, membrane and cytosolic fractions of the whole cell lysate were prepared for one replicate per condition of each tissue. To this end, the whole cell lysate was subjected to ultracentrifugation at 100,000 × g for 1 h at 18°C (TLA 100.3 rotor, Beckman Coulter). The supernatant (cytosolic fraction) and the dissolved pellet (membrane fraction; in 2% SDS lysis buffer) were subjected to protein precipitation as described above.

### Pan-mouse library generation, DIA-MS, and data analysis

Sample preparation, pan-mouse spectral library generation, and DIA-MS were performed by Biognosys (Zurich, Switzerland) as previously reported (Bruderer et al., 2015, 2017b; Rouwette et al., 2016). Data were analyzed with Spectronaut Pulsar (Biognosys) with precursor and protein FDR set at 0.01. Further analyses were performed with R (https://www.r-project.org/). Upon publication, all DIA-MS data, and the pan-mouse library will be publicly available in the Peptide Atlas (www.peptideatlas.org) and in our database painproteome.em.mpg.de.

#### Examining reproducibility

To examine reproducibility across samples, regression analyses were performed. Protein intensities were calculated per protein per replicate, as a mean intensity across peptides. Pearson's correlation coefficients were subsequently calculated for samples within each tissue, and kernel density estimates were used to visualize sample distribution. Initial analyses were performed with R, and subsequent plotting done with Python (Python Software Foundation, https://www.python.org/). Unsupervised hierarchical clustering was performed in Perseus v1.5.6.0 (Tyanova et al., 2016). The clustering was done with Euclidean distances and average linkage on mean protein intensities (per replicate), which had been log_2_ transformed and z-score normalized. For visualization purposes (i.e., to help find proteins showing similar expression), row clusters with an arbitrary chosen distance threshold of 3.4 were presented in different colors.

#### Establishing tissue proteomes

For each control (Sham) tissue, peptide intensities were combined across all three replicates for a given protein identifier. Mean intensity, standard deviation, and the number of peptides were calculated. A positive protein detection refers to at least one peptide in all three Sham replicates. We opted to include all proteins meeting these criteria (i.e., including proteins only identified by a single peptide across all three Sham replicates) to be able (i) to provide the most comprehensive analysis of tissue proteomes and (ii) to perform the most stringent comparisons across tissues possible (see below for the definition of tissue-enriched proteomes). To prevent redundancy in our protein list, as well as facilitate literature comparisons (described below), only protein identifiers corresponding to reviewed Uniprot entries (http://www.uniprot.org) were used for subsequent analysis. Additionally, detected keratin contamination was removed from the analysis. This analysis was repeated using a spectral library designed *in silico* from the mouse brain proteome data published by Sharma and colleagues (Sharma et al., 2015).

#### Quantification of changes in protein abundance upon SNI

To calculate expression changes in the SNI model, log_2_ intensity ratios were first calculated between SNI and Sham tissues at a peptide level. For each peptide, three ratios were calculated: SNI Replicate 1/Sham Replicate 1, SNI Replicate 2/Sham Replicate 2, SNI Replicate 3/Sham Replicate 3. Hence, peptides were only considered if they are detected across all three Sham replicates and all three SNI replicates. Mean log_2_ ratios were subsequently calculated for each protein ID, and one-sample *t*-tests were performed. After filtering for reviewed Uniprot IDs, *p*-values were adjusted using the Benjamini-Hochberg (BH) procedure (R: *p.adjust, method* = “*BH”*). Regulated proteins were defined as having a BH-adjusted *p*-value *q* < 0.05. If the regulation of a protein could only be established based on a single peptide across all six samples, it was considered a “low confidence regulated hit” and is highlighted as such in Supplementary Table 8 (according to latest considerations regarding the quantitation based on single-peptide hits, please see tutorial at http://www.mcponline.org/site/misc/ParisReport_Final.xhtml). In keeping with the Sham data, only reviewed Uniprot entries were considered and keratin contaminations were removed.

### Comparative proteome data analysis

#### Establishing a PNS-enriched proteome

In the interest of establishing a PNS-enriched proteome, DRG and SN data were compared to four brain tissues (ACC, AMY, PFC, and CER). A protein was considered to be PNS-enriched if either criteria was met: (A) protein was detected in DRG and/or SN but none of the brain tissues, or (B) 10-fold enrichment (one order of magnitude) in at least one PNS tissue compared to brain regions. To calculate the latter, protein abundance was compared between each PNS-tissue and the maximum abundance detected in a brain region. A 1-tailed Welch's *t*-test was used to determine significance (*p* < 0.05). Only proteins which were more than 10-fold enriched with a *p* < 0.05 were considered PNS-enriched for further analysis. To strengthen our confidence in the PNS-enriched proteome, we then compared this initial PNS-enriched list to data analyzed using the *in silico* library designed from the mouse brain proteome published by Sharma and colleagues (Sharma et al., 2015). Any protein which was detected in a brain tissue of this secondary analysis was removed from the PNS-enriched list, unless it met our 10-fold enrichment criteria. Again, proteins identified by a single peptide across replicates were included to enable the most stringent tissue comparison possible.

#### Establishing an SC-enriched proteome

An SC-enriched proteome was produced by mirroring the aforementioned criteria: non-detected in any brain region or 10-fold enriched (*p* < 0.05), with subsequent comparison to data analyzed using the *in silico* library designed from the mouse brain proteome data published by Sharma and colleagues (Sharma et al., 2015).

#### Published literature comparisons

To assess the quality of our datasets, as well as the translational applications, our data were compared to relevant previously published genomic, transcriptomic, and proteomic datasets. For each comparison, identifiers were uploaded to Uniprot and converted to reviewed mouse Uniprot identifiers. This conversion provided a framework for genomic and transcriptomic, as well as human data to be matched to our mouse-generated proteome. Of note, the conversion to reviewed Uniprot identifiers from previously published data resulted in a loss of some identifiers, occasionally reducing the total number of proteins used for the comparison. Even so, this standardization allowed for a comparison not otherwise possible. Percentage overlap was calculated between these lists and our datasets (e.g., all proteins, PNS-enriched, SC-enriched, proteins regulated in SNI/Sham, etc.). For comparison to the Human Protein Atlas (Uhlen et al., 2015; Thul et al., 2017), data were accessed in July 2017, corresponding to Protein Atlas version 16.1. Both “approved” and “supported” proteins at expression scores of “low,” “medium,” and “high” were included. Full details about the annotations can be found here: https://www.proteinatlas.org/about/assays+annotation. In essence, reliably detected proteins were examined across all tissues. Proteins with uncertain reliability scores and those “not detected” were excluded for a given tissue. All tissues were compiled for the analysis, as no data from PNS tissue was included in the Atlas. Identifiers were then uploaded to Uniprot and converted, as described above.

#### Retrospective analysis of two chronic pain models

Previously, our group has reported changes in the DRG membrane proteome upon SNI and inflammatory pain induced by CFA injection (Rouwette et al., 2016). These data were reanalyzed with our updated generated pan-mouse library. Proteins regulated in either pain model were subsequently compared to determine commonly-regulated proteins.

#### Ingenuity pathway analysis (IPA®)

Protein lists (per tissue: Sham and SNI/Sham, PNS-enriched, SC-enriched) were uploaded to IPA® (Qiagen) for further analysis. For SNI/Sham data, only significantly regulated proteins *q* < 0.05 (BH-adjusted *p*-value) were considered.

#### Gene ontology analysis

Protein lists (per tissue: Sham and SNI/Sham, PNS-enriched, SC-enriched) were uploaded to the web interfaces DAVID (david.ncifcrf.gov; Huang da et al., 2009) and STRING (string-db.org; Franceschini et al., 2013). We reported only significant enrichments (FDR < 0.05) in cellular component (CC) or biological process (BP).

## Results

### Standardized proteome profiling of tissues relevant for pain

The goal of our study was twofold. On one hand, we aimed to comprehensively define the adult mouse proteome of the SN, DRG, and SC. On the other hand, we envisioned using this information to enable the trustful monitoring of proteome alterations during a chronic pain condition. To this end, we chose the widely employed spared-nerve-injury (SNI)-model of neuropathic pain (Decosterd and Woolf, 2000) and performed a comparison to Sham-operated control mice (please see Methods for details on mice).

**Figure 1:**
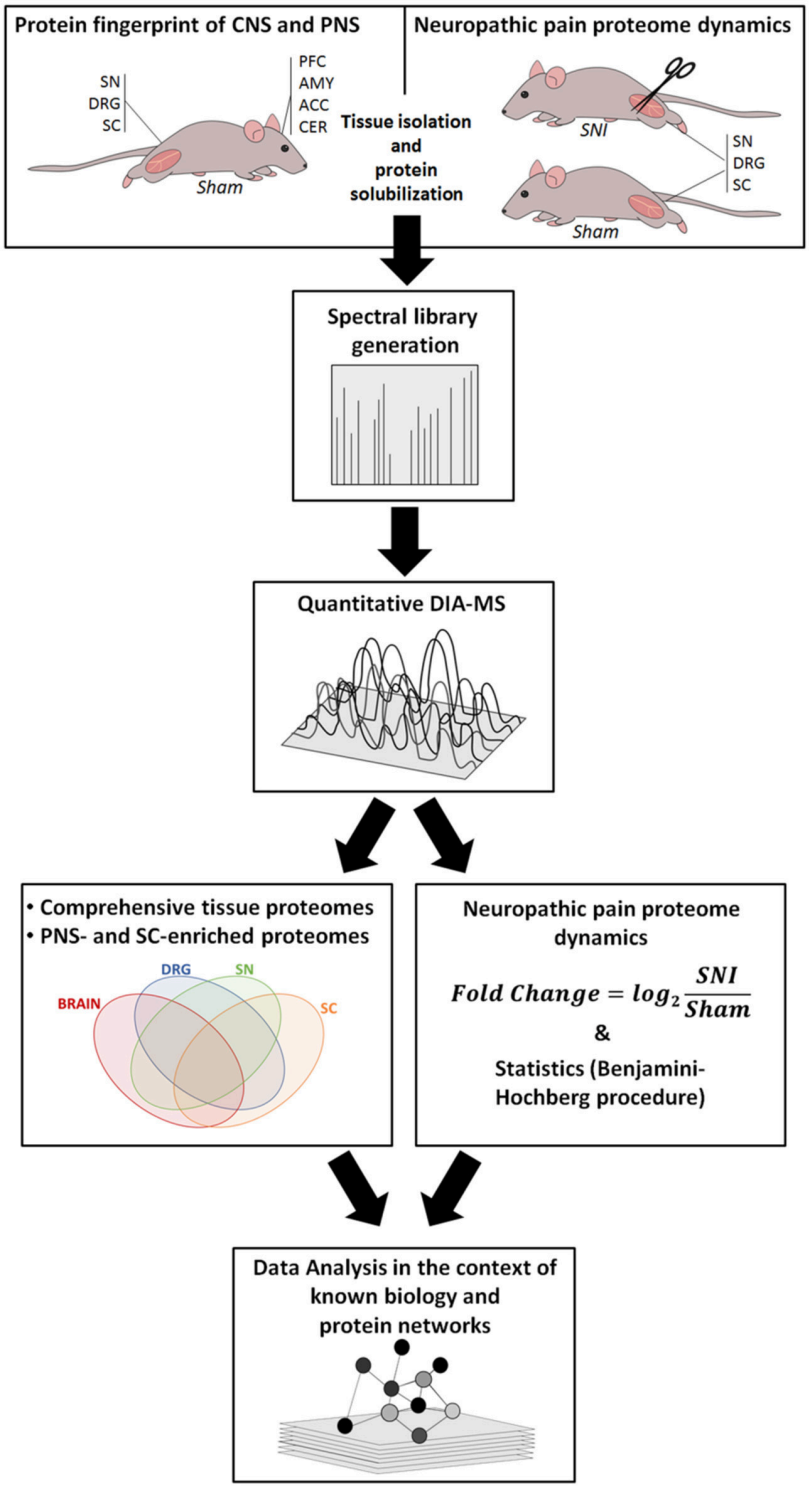
Overview of our integrated workflow: from Sham-operated mice and the SNI model via mouse behavior, protein solubilization, quantitative DIA-MS and data analysis to protein network dynamics. ACC, anterior cingulate cortex; AMY, amygdala; CER, cerebellum; DRG, dorsal root ganglia; PFC, prefrontal cortex; SC, spinal cord; SN, sciatic nerve.

Our workflow is outlined in Figure 1, and ranges from the induction of neuropathic pain, via DIA-MS to an in-depth data and pathway analysis.

Initially, we focused on Sham-operated mice as controls to define region-resolved proteomes (Figure 1 left). We prepared lysates of the SN, DRG, and SC. For the sake of comparative analysis between the PNS (SN and DRG) and CNS, we additionally prepared lysates of four major brain regions: the prefrontal cortex (PFC), the anterior cingulate cortex (ACC), the amygdala (AMY), and the cerebellum (CER). The first three CNS regions were chosen based on their implication in chronic pain states. The CER was chosen based on a recent study showing that the DRG transcriptome resembled the cerebellum more than any other brain region (Parisien et al., 2017).

Using mass spectrometry, we first generated a spectral library according to previously established procedures (Rouwette et al., 2016; Figure 1). Next, we assembled a pan-mouse peptide spectral library (pan-mouse library) by combining individual tissue libraries obtained in this study with our previously published libraries (Rouwette et al., 2016; Bruderer et al., 2017a,b). Taken together, this pan-mouse library contains peptides corresponding to 6205 proteins (complete list: Supplementary Table 1). These include 3008 proteins predicted to be localized to the membrane according to gene ontology (GO) analysis for cellular compartment, GO_CC (BH-corrected *p*-value: 5,2 × 10^−173^) using the web interface DAVID (Huang da et al., 2009). Among these, 254 are annotated to be involved in ion transport (GO analysis for biological process, GO_BP; BH-corrected *p*-value: 1,8 × 10^−7^) including ion channels and receptors with prominent functions in nociceptive processing, e.g., voltage-dependent calcium (Ca_v_) and sodium (Na_v_) channel subunits, members of the transient receptor potential (TRP) family, and glutamate receptors (complete list: Supplementary Table 1). We have recently shown that mouse spectral libraries can be applied for protein profiling across species, i.e., also in humans (Bruderer et al., 2017b). Based on its comprehensive protein coverage, our pan-mouse library can be used for the standardized interrogation of diverse mouse and human datasets in pain research. It contains information of 31% of the genes represented in the Human Pain Genes Database (HPGDB, https://humanpaingenetics.org/hpgdb/; Meloto et al., 2017) as well as 45% of genes identified in pain-related transgenic knockout studies summarized in the Pain Genes Database (PGD, http://www.jbldesign.com/jmogil/enter.html; Lacroix-Fralish et al., 2007; complete list: Supplementary Table 1). Therefore, our pan-mouse library represents a valuable resource to profile these pain-related proteins and others in human and rodent tissues.

**Figure 2:**
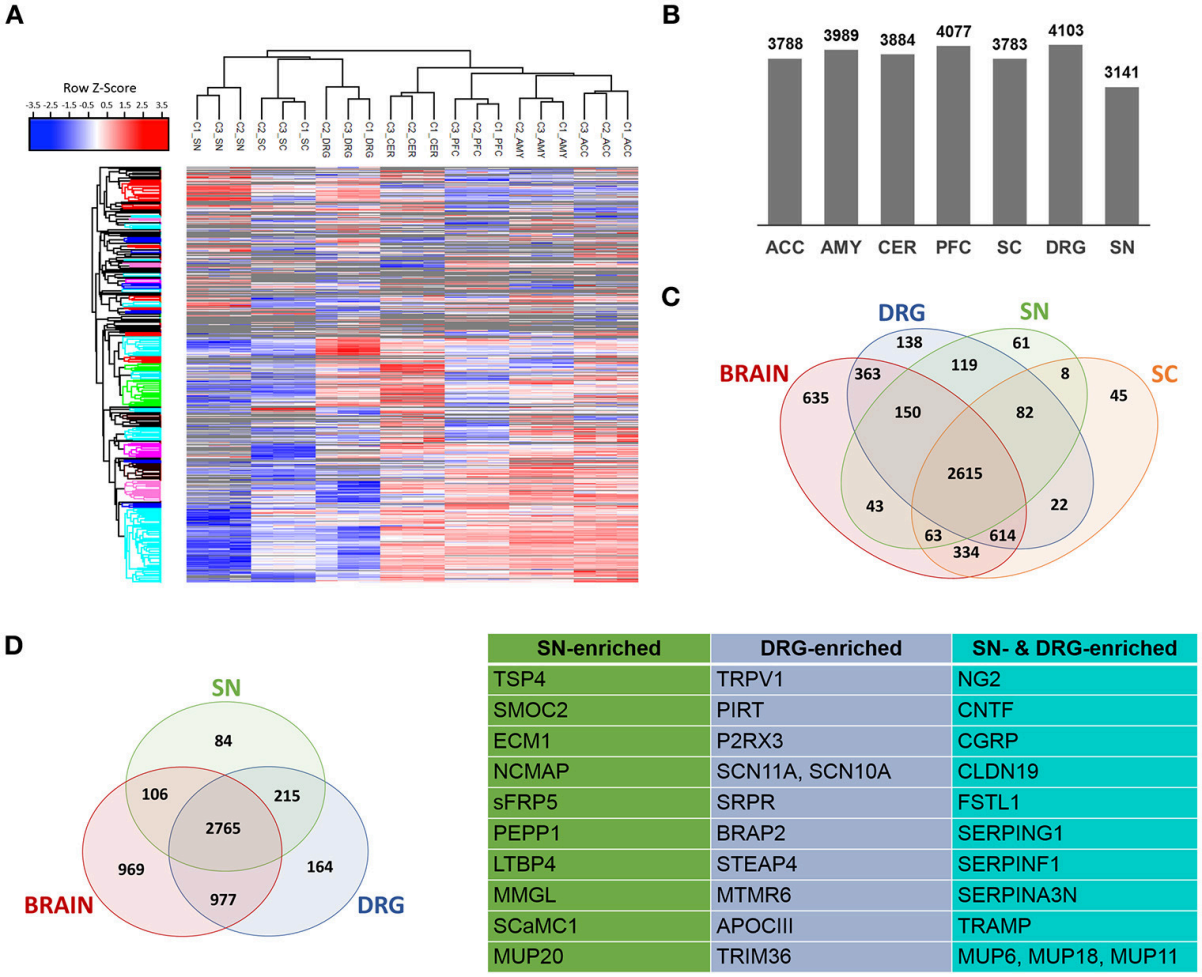
Determination of PNS- and SC-enriched proteomes. **(A)** Unsupervised clustering of z-scored normalized protein intensities from all Sham replicates (C1-C3; 4 mice each) across the 7 analyzed regions of the PNS and CNS. High experimental reproducibility becomes apparent. Furthermore, the clustering shows expected similarities between related tissues, e.g., PNS tissues and spinal cord (SC) are more closely clustered together than the 4 brain regions. The different colors of the y-axis dendrogram visualize exemplary clusters of proteins. **(B)** Number of proteins consistently detected across tissues. **(C)** Venn diagram illustrates differentially expressed and shared proteins across SN, DRG, SC, and brain regions. **(D)** A quantitative comparative analysis reveals that 299 and 379 proteins are enriched (> 10-fold) in SN and DRG proteomes (i.e., PNS-enriched) compared to the brain proteome. Further, tissue-specific expression of 84 (SN) and 164 (DRG) proteins can be observed. The table lists examples of PNS-enriched proteins according to their expression in SN, DRG, or both.

Next, we performed DIA-MS experiments according to published procedures (Figure 1 workflow; Bruderer et al., 2015, 2017a,b). This enabled us to quantify >5,000 proteins across DIA-MS runs. Applying very stringent criteria for reproducibility of protein identification across all replicates (please see Methods for details) resulted in highly reproducible profiling of 3141-4103 proteins depending on the tissue (Figures 2A–C; Supplementary Figure 2; complete list: Supplementary Table 2). While the vast majority of proteins were expressed across regions, differentially expressed proteins could be determined when comparing the brain proteome with SC and PNS proteomes (Figure 2C; complete list: Supplementary Table 2). Among the latter, we observed a significant overlap of SC and DRG proteomes (Figure 2C), which is anatomically supported given that spinal roots (central branches of DRG axons) innervate the SC dorsal horn. Hierarchical clustering across individual replicates further supports the similarity of the DRG and SC proteomes (Figure 2A). Together, this data prompted us to analyze the SC separately from other CNS (i.e., brain) areas.

We then performed pathway analysis with IPA® to determine enriched canonical and disease pathways in each PNS, SC, and brain region analyzed. IPA® results recalled known global and region-specific features, demonstrating the quality and biological significance of identified proteomes (complete list: Supplementary Table 3). Among the top pathways highly enriched in the PNS compared to brain areas were *acute phase response signaling, LXR/RXR (liver x receptors/retinoid x receptors) activation*, the *complement system*, and the *superpathway of cholesterol biosynthesis*. Proteins belonging to these pathways are indicated in Supplementary Table 3. Conversely, *glutamate, GABA*, and *dopamine receptor signaling*, as well as *synaptic long term potentiation/depression* were found to be enriched in analyzed brain tissues as expected. Moreover, disease/functional pathways such as *cognition, learning, seizures, plasticity of synapse, locomotion*, and *schizophrenia* were highly represented in brain tissues compared to tissues of the PNS (complete list: Supplementary Table 3). In contrast, PNS and SC proteomes showed enrichment of proteins involved in *demyelinating neuropathy, fatty acid metabolism, myopathy*, and *chronic skin disorder*. Within the PNS we detected differences attributable to the diverse biology of SN and DRG, such as the overrepresentation of proteins associated with *(de)myelination, psoriasis*, and *chronic inflammatory disorder*, as well as *extracellular matrix* and *adhesion* in the SN vs. DRG.

### Defining discrete protein enrichment in PNS and SC proteomes

To objectively define PNS- and SC-enriched proteomes, we performed a quantitative and subtractive cross-comparison with the identified brain proteome, applying stringent selection criteria (please see Methods for details). First, protein enrichment in the PNS or SC was defined by > 10-fold enrichment of protein abundance. As a second filter, we used data published by Sharma and colleagues (Sharma et al., 2015), which represents the most extensive mouse brain proteome to date. We then again excluded every protein from our PNS or SC-enriched protein compendium that was not >10-fold enriched over any brain tissue using the *in silico* library designed from data published by Sharma and colleagues.

This procedure yielded the definition of 463 PNS-enriched proteins, 164 of which were preferably expressed in DRG and 84 in SN (Figure 2D; complete list: Supplementary Table 4). Among these proteins are several well-known key PNS proteins, confirming the validity of our results. These include hallmark ion channels of sensory neurons, such as the voltage-gated sodium channels SCN11A and SCN10A, the TRP ion channel TRPV1 and its interaction partner PIRT, and the purinergic ion channel P2RX3. Moreover, the SN proteome featured prominent myelin proteins, e.g., proteolipidprotein 2 (PLP2), peripheral myelin protein 2 (PMP2) and NCMAP, as well as several other candidates with reported involvement in pain or PNS neurobiology: thrombospondin-4 (TSP4), ciliary neurotrophic factor (CNTF), Calcitonin gene-related peptide 1 (CGRP), follistatin 1 (FSTL1), and the Schwann cell tight junction protein claudin 19 (CLDN19; Miyamoto et al., 2005; Li et al., 2011a,b; Kim et al., 2012). The latter four are shared among DRG and SN.

More interestingly, we detected many proteins which are hardly characterized within the PNS. To illustrate, the tripartite motif-containing 36 protein (TRIM36) is an E3 ubiquitin-protein ligase which may bind microtubules and is involved in cell cycle regulation (Miyajima et al., 2009). Interestingly, its transcript has been found to be enriched in mouse and human DRG (Ray et al., 2018). We further identified C-type lectin domain family 10A (MMGL; Zizzari et al., 2015), which acts as an immune system modulator, particularly during inflammation. Members of the serpin family of serine protease inhibitors such as SERPINA3N, SERPING1, and SERPINF1 were also found to be enriched in the PNS. SERPINs are known to exhibit multiple functions from controlling the complement system to acute inflammatory immune responses (Gettins, 2002). SERPINF1 is notable as it lacks the protease inhibitory activity and is known to be antiangiogenic and neurotrophic (Gettins et al., 2002; Rychli et al., 2009). The role of SERPING1 and F1 in the PNS is unknown, but worthwhile investigating: Vicuna and colleagues have recently shown the prominent role of DRG-derived SERPINA3N in promoting early phases of neuropathic pain upon SNI (Vicuna et al., 2015).

Another prominent group, which is enriched in SN and has some overlap with the DRG, are proteins associated with the extracellular matrix (ECM). Among the enriched ECM proteins in our PNS-resolved proteomes, we identified: Chondroitin sulfate proteoglycan 4 (NG2; Fukushi et al., 2004), known to contribute to cell migration, neurite outgrowth and angiogenesis; latent transforming growth factor beta binding protein 4 (LTBP4; Lamar et al., 2016), involved in muscular dystrophy through the activity control of TGF-beta; dermatopontin (TRAMP; Krishnaswamy et al., 2014), a stabilizer of collagen fibrils facilitating wound healing; SPARC related modular calcium binding 2 (SMOC2; Vannahme et al., 2003), implicated in angiogenesis and matrix assembly facilitating wound healing; and extracellular matrix protein 1 (ECM1; Anderson et al., 2009) with functions in tumor biology, skin integrity, and inflammatory bowel disease.

We performed a similar analysis to define an SC-enriched proteome (in comparison to the brain proteome) yielding 74 proteins (complete list: Supplementary Table 5). The SC-enriched proteome contains the purinergic ion channel P2RX7 and other proteins known to be involved in nociception or pain, including Fibroblast Growth Factor Receptor 1 (FGFR-1) and S100 Calcium Binding Protein A8 (S100A8; Jamieson et al., 2014). Like in our PNS-enriched list, proteins with largely unexplored function in the SC can also be found. These include: Tax1-binding protein 3 (TIP-1; Kanamori et al., 2003) with effects on Wnt-signaling via catenin inhibition; microfibril-associated glycoprotein 4 (MFAP4; Koyanagi et al., 2016), an extracellular integrin ligand with glucocorticoid-dependent diurnal oscillations in the SC; and zinc finger protein (DPF3; Lange et al., 2008) implicated in developmental chromatin remodeling.

### Quality of resolved PNS and SC proteomes

To determine the quality of our analysis, we assessed various aspects of resolved PNS and SC proteomes. Considering the importance of membrane proteins as drug targets (Dib-Hajj et al., 2009; Patapoutian et al., 2009; Lunn, 2010; Raouf et al., 2010; Antunes-Martins et al., 2013; Jamieson et al., 2014), we performed a GO-based enrichment analysis for cellular compartment using the web interface DAVID (Huang da et al., 2009). This revealed that membrane proteins are well-represented in these proteomes: membrane proteins constitute roughly 50% of proteins across analyzed proteomes, approximately 40% of which are predicted to be localized to the plasma membrane (PM) [SC: 50% (1897/3779) of which 40% (764/1897) at PM; DRG: 49.2% (2017/3817) of which 37.8% (764/2017) at PM; SN: 49.5% (1551/3136) of which 40.0% (621/1551) at PM].

**Figure 3:**
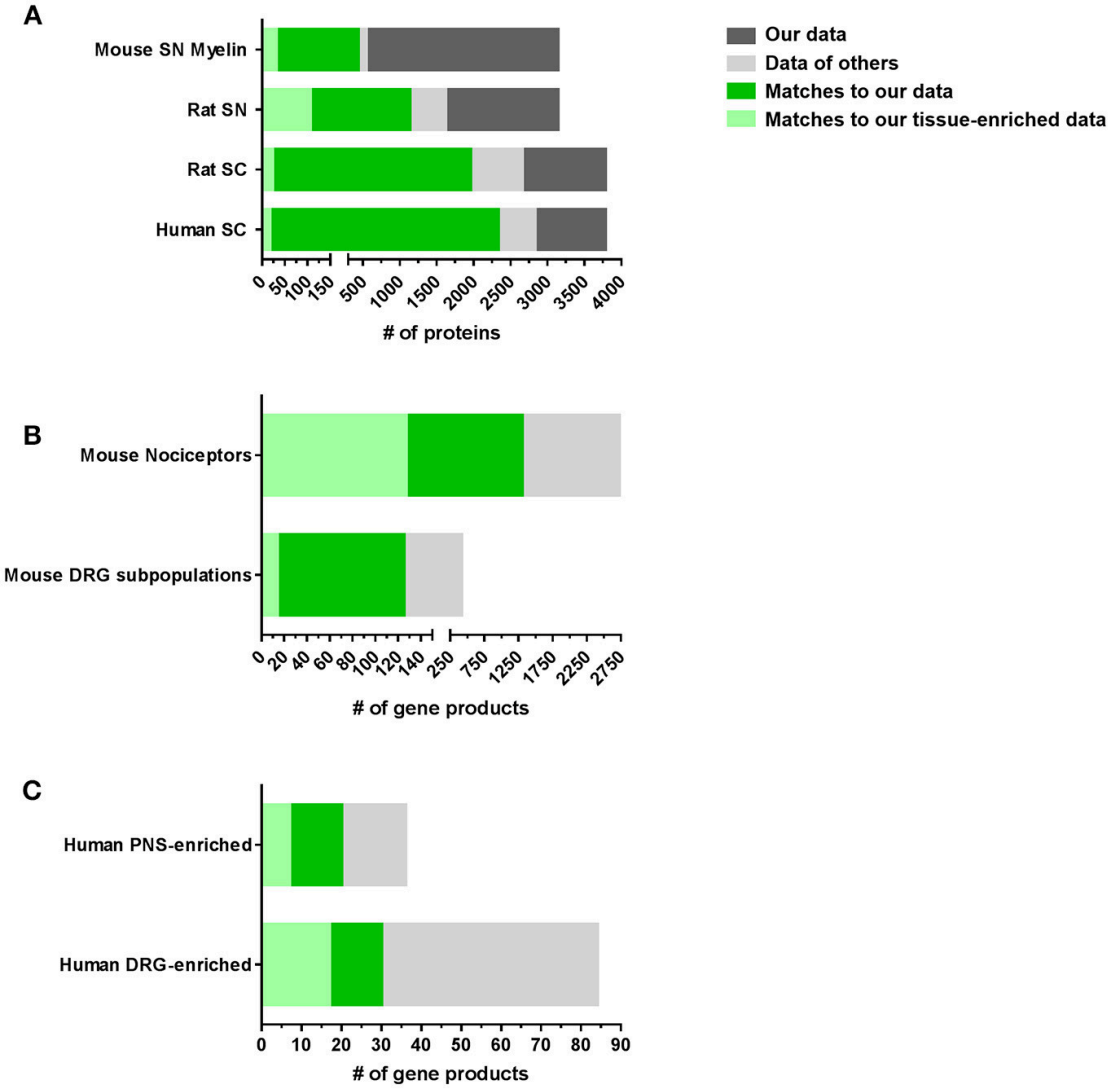
Comparison of PNS and SC proteomes with previously published reports in mice and humans. **(A)** Comparison of our SN or SC proteomes with published proteome studies: SN mouse myelin proteome (Patzig et al., 2011), SN rat membrane proteome (Lu et al., 2009), SC rat membrane proteome (Lu et al., 2009), and SC human proteome (Bruderer et al., 2017b). **(B)** Comparison of our DRG proteomes with published DRG transcriptome/RNA-seq studies: mouse DRG nociceptors (Thakur et al., 2014) and mouse DRG neuronal subpopulations (Usoskin et al., 2015). **(C)** Comparison of our PNS and DRG proteomes with studies on the human PNS-enriched transcriptome (Sapio et al., 2016) and the human DRG-enriched transcriptome (Ray et al., 2018).

Next, we compared our datasets with published reports on the molecular composition of the PNS and SC (complete list: Supplementary Table 6). With few published proteome studies on these tissues, we also included transcriptome data from DRG, SN, and SC in our comparison. However, comparisons between transcriptomes and proteomes often show major discrepancies due to the well-known limited predictability of mRNA alterations for protein levels (Schwanhausser et al., 2011; Sharma et al., 2015; Liu et al., 2016). These limitations need to be considered when interpreting data. Nonetheless, our results demonstrate a significant overlap with previous -omics studies on SC, SN, and DRG essentially validating our datasets (Figure 3A). Our SN proteome comprises >70% of the published rat SN membrane proteome (Lu et al., 2009) and the mouse myelin proteome (Patzig et al., 2011). Moreover, overlapping proteins represent 10% (31/299) and 35% (106/299) of the SN-enriched proteome as defined above, respectively. A comparison of our SC data with the published rat SC membrane proteome (Lu et al., 2009) and the published human SC proteome (Bruderer et al., 2017b) yielded similar results, i.e., strong overlaps of >70% (Figure 3A; complete list: Supplementary Table 6).

Remarkably, we also observed pronounced similarities between the DRG proteome identified here and previous mRNA-based studies on purified nociceptors (Thakur et al., 2014) and sensory neuron subtypes of DRG (Usoskin et al., 2015; Figure 3B). For example, 31% (1306/4103) of all identified proteins in DRG and 33% (127/379) of DRG-enriched proteins match nociceptor mRNA (Thakur et al., 2014), of which we obtain a 47% (1306/2733) coverage on protein level (Figure 3B). Even more, our DRG proteome list contains information on 29% (125/417 annotated mRNAs) of the characteristic top 50 members across most DRG subpopulations (Usoskin et al., 2015; Figure 3B) including neurofilament-rich myelinated neurons (NF), peptidergic nociceptors (PEP), nonpeptidergic nociceptors (NP), and tyrosine hydroxylase-expressing neurons (TH; results for neuronal subpopulations: Supplementary Figure 3 and Supplementary Table 6). Given that we used lysates of whole DRG containing glia and immune cells alongside neurons, this analysis strongly suggests that a substantial part of our dataset represents the neuronal proteome of DRG. The lists of all comparisons and overlapping proteins can be found in Supplementary Table 6. These findings validate our results and support their significance for further hypothesis-driven analysis at the protein level across cell types. They also highlight their utility for discovering uncharacterized neuronal and non-neuronal proteins.

### Utility of our datasets for translational pain research

The motivation underlying high-throughput methodologies is to identify novel molecular and mechanistic insights into complex pathological processes. Therefore, we aimed at critically examining whether our proteome datasets could also provide a resource to explore the molecular set-up of painful pathologies across species to enable translational work. First, we investigated the coverage of genes and gene products reported to underlie painful pathologies in humans and rodents. Again, we leveraged previous data mining efforts and compared our data with the Human Pain Gene Database [HPGDB, (Meloto et al., 2017)] and with pain-related transgenic knockout studies summarized in the Pain Genes Database [PGD, (Lacroix-Fralish et al., 2007)] (complete list: Supplementary Table 7). Indeed, many pain-related gene products are represented in our proteome datasets, strongly supporting the utility of our data for investigating pathological processes associated with pain: specifically, our PNS proteome includes 17% of the HPGDB (23/129; 9 defined as PNS-enriched) and 30% of the PGD (133/435; 31 defined as PNS-enriched ones). Similar values were obtained for the SC proteome, with 16% of the HPGDB (21/129; 2 defined as SC-enriched) and 26% of the PGD (116/435; 2 defined as SC-enriched).

Considering that side effects on key bodily functions limit current therapeutic options of many painful disorders, defining a PNS-enriched set of proteins in a body-wide manner is of immense importance (Patapoutian et al., 2009; Borsook et al., 2014; Berta et al., 2017b; Ray et al., 2018). Excellent recent studies have systematically investigated this on the transcriptome level by comparing human and mouse datasets (Sapio et al., 2016; Ray et al., 2018). They reported a PNS-enriched and DRG-enriched transcriptome, respectively, which is conserved between mouse and humans. Intriguingly, we observed a significant overlap with our PNS-enriched datasets (complete list: Supplementary Table 7): Our full PNS proteome comprises 75% (27/36) of reported human PNS-enriched gene products (Sapio et al., 2016) including 19% (7/36) in our list of PNS-enriched proteins, such as PNS hallmark ion channels SCN11A, SCN10A, and TRPV1 (Figure 3C). Moreover, among the 84 conserved and DRG-enriched transcripts described by Ray and colleagues (Ray et al., 2018), we detected 35% (30/84) at the protein level in DRG. Of these 30 proteins, 17 match to our DRG-enriched dataset and 20 to our PNS-enriched dataset including aforementioned hallmark proteins of the PNS (Figure 3C; complete list: Supplementary Table 7).

Despite these matches, we recognize that our proteome data (i) lacks information on 65% (54/84 reported by Ray and colleagues; Ray et al., 2018) of conserved and DRG-enriched transcripts, and (ii) on the other hand, does not confirm the DRG-enrichment of others at the protein level. To illustrate the latter, we identified 13 proteins in our brain proteome (Supplementary Table 2), including stathmin 2 (STMN2), tubulin-beta 3 (TUBB3), collagen 28 (COL28A1), protein prune homolog 2 (PRUNE2), and known DRG marker proteins like advillin (AVIL) and peripherin (PRPH; complete list: Supplementary Table 7). Reasons for the missing proteins in our data are likely related to well-known limitations of proteomics approaches in detecting low abundant proteins in complex samples; notably for transmembrane proteins. On the other hand, many studies have demonstrated a prominent mismatch between transcriptome and proteome data due to posttranslational modifications, altered protein stability, and cellular buffering mechanisms (Schwanhausser et al., 2011; Sharma et al., 2015; Liu et al., 2016). Hence, mismatches in defining protein-enrichment may arise.

To address this, in part, we extended our definition of PNS-enriched proteins with respect to their body-wide expression pattern in humans at the protein level. To this end, we employed the exhaustive immunohistochemistry-based dataset available in the Human Protein Atlas (Uhlen et al., 2015; Thul et al., 2017), which contains protein expression data on 30 different tissues, but excludes the PNS and SC. We scrutinized our mouse PNS-enriched proteome data for those proteins that did not match the Human Protein Atlas. Notably, we uncovered 31% (142/463) as putative PNS-enriched candidate proteins in humans given the previously shown good agreement between sensory neurons in mice and humans (Sapio et al., 2016; Lopes et al., 2017; Ray et al., 2018). Validating our results, this list contains well known PNS proteins (TRPV1, PIRT, SCN11A, SCN10A, P2RX3, CLDN19, CNTF) as well as diverse myelin proteins of the SN, like noncompact myelin-associated protein (MP11), myelin P2 protein (MYP2), and myelin protein P0 (MYP0; complete list: Table 1). The list also encompasses 14 candidates reported by Ray and colleagues (Ray et al., 2018) to be DRG-enriched and conserved in humans (labeled with ^*^ in Table 1).

**Table 1:** List of putative PNS-enriched candidate proteins in humans.

**Entry**	**Protein names**	**Gene names**
Q9WV68	Peroxisomal 2,4-dienoyl-CoA reductase (EC 1.3.1.34) (2,4-dienoyl-CoA reductase 2)	Decr2 Pdcr
Q9WUP4	Polyprenol reductase (EC 1.3.1.94) (3-oxo-5-alpha-steroid 4-dehydrogenase 3) (EC 1.3.1.22) (Steroid 5-alpha-reductase 2-like) (Steroid 5-alpha-reductase 3) (S5AR 3) (SR type 3)	Srd5a3 Srd5a2l
Q9WU66^*^	Secreted frizzled-related protein 5 (sFRP-5)	Sfrp5
Q9R059	Four and a half LIM domains protein 3 (FHL-3) (Skeletal muscle LIM-protein 2) (SLIM-2)	Fhl3
Q9R053^*^	Sodium channel protein type 11 subunit alpha (NaN) (Sensory neuron sodium channel 2) (Sodium channel protein type XI subunit alpha) (Voltage-gated sodium channel subunit alpha Nav1.9)	Scn11a Nan Nat Sns2
Q9QZZ6	Dermatopontin (Early quiescence protein 1) (EQ-1) (Tyrosine-rich acidic matrix protein) (TRAMP)	Dpt
Q9QYY0	GRB2-associated-binding protein 1 (GRB2-associated binder 1) (Growth factor receptor bound protein 2-associated protein 1)	Gab1
Q9JLK7	Calcium-binding protein 1 (CaBP1)	Cabp1
Q9JLB2	MAGUK p55 subfamily member 5 (Protein associated with Lin-7 1)	Mpp5 Pals1
Q9JKJ9	24-hydroxycholesterol 7-alpha-hydroxylase (EC 1.14.14.26) (Cytochrome P450 39A1) (mCYP39A1) (Oxysterol 7-alpha-hydroxylase)	Cyp39a1
Q9JJH1	Ribonuclease 4 (RNase 4) (EC 3.1.27.-)	Rnase4
Q9ET38^*^	Claudin-19 (CLDN19)	Cldn19
Q9ESB3	Histidine-rich glycoprotein (Histidine-proline-rich glycoprotein) (HPRG)	Hrg
Q9ERA0	Alpha-globin transcription factor CP2	Tfcp2 Tcfcp2
Q9ER60	Sodium channel protein type 4 subunit alpha (Sodium channel protein skeletal muscle subunit alpha) (Sodium channel protein type IV subunit alpha) (Voltage-gated sodium channel subunit alpha Nav1.4)	Scn4a
Q9EPX2	Papilin	Papln
Q9EPV8	Ubiquitin-like protein 5	Ubl5
Q9EPL9	Peroxisomal acyl-coenzyme A oxidase 3 (EC 1.3.3.6) (Branched-chain acyl-CoA oxidase) (BRCACox) (Pristanoyl-CoA oxidase)	Acox3
Q9EPB5	Serine hydrolase-like protein (SHL) (EC 3.1.-.-)	Serhl
Q9DBN5	Lon protease homolog 2, peroxisomal (EC 3.4.21.-) (Lon protease-like protein 2) (Lon protease 2) (Peroxisomal Lon protease)	Lonp2
Q9DBG7	Signal recognition particle receptor subunit alpha (SR-alpha) (Docking protein alpha) (DP-alpha)	Srpra Srpr
Q9DBD0	Inhibitor of carbonic anhydrase	Ica
Q9DB16	Calcium-binding protein 39-like (MO25beta) (Mo25-like protein)	Cab39l
Q9D2R4	Transmembrane emp24 domain-containing protein 11 (Glycoprotein 25L) (GP25L) (p24 family protein alpha-1) (p24alpha1)	Tmed11
Q9D281	Protein Noxp20 (Nervous system overexpressed protein 20) (Protein FAM114A1)	Fam114a1 Noxp20
Q9CXX9	CUE domain-containing protein 2	Cuedc2
Q9CWP6	Motile sperm domain-containing protein 2	Mospd2
Q9CWG8	Protein arginine methyltransferase NDUFAF7, mitochondrial (EC 2.1.1.320) (NADH dehydrogenase [ubiquinone] complex I, assembly factor 7) (Protein midA homolog)	Ndufaf7
Q9CQW9	Interferon-induced transmembrane protein 3 (Dispanin subfamily A member 2b) (DSPA2b) (Fragilis protein) (Interferon-inducible protein 15) (Mouse ifitm-like protein 1) (Mil-1)	Ifitm3
Q9CQ20	Mid1-interacting protein 1 (Gastrulation-specific G12-like protein) (Mid1-interacting G12-like protein) (Protein STRAIT11499 homolog) (Spot 14-related protein) (S14R) (Spot 14-R)	Mid1ip1 Mig12
Q99PM3	Transcription initiation factor IIA subunit 1 (General transcription factor IIA subunit 1) [Cleaved into: Transcription initiation factor IIA alpha chain (TFIIA p35 subunit); Transcription initiation factor IIA beta chain (TFIIA p19 subunit)]	Gtf2a1
Q99M08	Uncharacterized protein C4orf3 homolog	*NA*
Q99LJ6	Glutathione peroxidase 7 (GPx-7) (GSHPx-7) (EC 1.11.1.9)	Gpx7
Q99JS0^*^	Noncompact myelin-associated protein (Myelin protein of 11 kDa) (MP11)	Ncmap
Q99JF5	Diphosphomevalonate decarboxylase (EC 4.1.1.33) (Mevalonate (diphospho)decarboxylase) (MDDase) (Mevalonate pyrophosphate decarboxylase)	Mvd
Q99JA0^*^	Calcitonin gene-related peptide 1 (Alpha-type CGRP) (Calcitonin gene-related peptide I) (CGRP-I)	Calca Calc
Q923B6	Metalloreductase STEAP4 (EC 1.16.1.-) (Dudulin-4) (Six-transmembrane epithelial antigen of prostate 4) (Tumor necrosis factor-alpha-induced adipose-related protein)	Steap4 Tiarp
Q921L5	Conserved oligomeric Golgi complex subunit 2 (COG complex subunit 2) (Component of oligomeric Golgi complex 2) (Low density lipoprotein receptor defect C-complementing protein)	Cog2 Ldlc
Q91YI4	Beta-arrestin-2 (Arrestin beta-2)	Arrb2
Q91WP6	Serine protease inhibitor A3N (Serpin A3N)	Serpina3n Spi2
Q91W59	RNA-binding motif, single-stranded-interacting protein 1 (Single-stranded DNA-binding protein MSSP-1)	Rbms1 Mssp Mssp1
Q91V01	Lysophospholipid acyltransferase 5 (LPLAT 5) (EC 2.3.1.-) (1-acylglycerophosphocholine O-acyltransferase) (EC 2.3.1.23)	Lpcat3 Grcc3f Mboat5 Oact5
Q8VE11	Myotubularin-related protein 6 (EC 3.1.3.-)	Mtmr6
Q8VC98^*^	Pleckstrin homology domain-containing family A member 4 (PH domain-containing family A member 4) (Phosphoinositol 3-phosphate-binding protein 1) (PEPP-1)	Plekha4 Pepp1
Q8R3U1	HRAS-like suppressor 3 (HRSL3) (EC 3.1.1.32) (EC 3.1.1.4) (Adipose-specific phospholipase A2) (AdPLA) (Group XVI phospholipase A2) (H-rev 107 protein homolog)	Pla2g16 H-rev107 Hrasls3 Hrev107
Q8R3I3	Conserved oligomeric Golgi complex subunit 6 (COG complex subunit 6) (Component of oligomeric Golgi complex 6)	Cog6 Kiaa1134
Q8R3I2	Lysophospholipid acyltransferase 2 (LPLAT 2) (EC 2.3.1.-) (1-acylglycerophosphocholine O-acyltransferase) (EC 2.3.1.23)	Mboat2 Lpcat4 Oact2
Q8R189	Monocyte to macrophage differentiation factor 2 (Progestin and adipoQ receptor family member X)	Mmd2 Paqr10
Q8R100	Protein FAM26E	Fam26e
Q8K4K6	Pantothenate kinase 1 (mPank) (mPank1) (EC 2.7.1.33) (Pantothenic acid kinase 1)	Pank1 Pank
Q8K2V1	Serine/threonine-protein phosphatase 4 regulatory subunit 1	Ppp4r1
Q8K296	Myotubularin-related protein 3 (EC 3.1.3.48) (Phosphatidylinositol-3,5-bisphosphate 3-phosphatase) (EC 3.1.3.95) (Phosphatidylinositol-3-phosphate phosphatase) (EC 3.1.3.64)	Mtmr3
Q8K1B8	Fermitin family homolog 3 (Kindlin-3) (Unc-112-related protein 2)	Fermt3 Kind3 Urp2
Q8CI59	Metalloreductase STEAP3 (EC 1.16.1.-) (Dudulin-2) (Protein nm1054) (Six-transmembrane epithelial antigen of prostate 3) (Tumor suppressor-activated pathway protein 6)	Steap3 Tsap6
Q8CG14	Complement C1s-A subcomponent (EC 3.4.21.42) (C1 esterase) (Complement component 1 subcomponent s-A) [Cleaved into: Complement C1s-A subcomponent heavy chain; Complement C1s-A subcomponent light chain]	C1sa C1s
Q8CD91	SPARC-related modular calcium-binding protein 2 (Secreted modular calcium-binding protein 2) (SMOC-2)	Smoc2
Q8C650	Septin-10	Sept10
Q8C008	Double zinc ribbon and ankyrin repeat-containing protein 1	Dzank1
Q8BWL5	RNA-binding motif, single-stranded-interacting protein 3	Rbms3
Q8BS95	Golgi pH regulator (Protein GPR89)	Gpr89a Gphr Gpr89
Q8BRH3	Rho GTPase-activating protein 19 (Rho-type GTPase-activating protein 19)	Arhgap19
Q8BND5	Sulfhydryl oxidase 1 (mSOx) (EC 1.8.3.2) (Quiescin Q6) (Skin sulfhydryl oxidase)	Qsox1 Qscn6 Sox
Q8BMB3	Eukaryotic translation initiation factor 4E type 2 (eIF-4E type 2) (eIF4E type 2) (eIF4E-2) (mRNA cap-binding protein type 2) (Eukaryotic translation initiation factor 4E-like 3) (eIF4E-like protein 4E-LP)	Eif4e2 Eif4el3
Q8BH97	Reticulocalbin-3	Rcn3 D7Ertd671e
Q8BGD6	Sodium-coupled neutral amino acid transporter 9 (Solute carrier family 38 member 9)	Slc38a9
Q8BFY0^*^	Phosphoinositide-interacting protein	Pirt
Q80X19	Collagen alpha-1(XIV) chain (COEA1)	Col14a1
Q80WG7^*^	E3 ubiquitin-protein ligase Trim36 (EC 2.3.2.27) (Acrosome RBCC protein) (Haprin) (RING-type E3 ubiquitin transferase TRIM36) (Tripartite motif-containing protein 36)	Trim36
Q80VM7	Ankyrin repeat domain-containing protein 24	Ankrd24
Q7TQ62	Podocan	Podn
Q704Y3^*^	Transient receptor potential cation channel subfamily V member 1 (TrpV1) (Osm-9-like TRP channel 1) (OTRPC1) (Vanilloid receptor 1)	Trpv1
Q6QIY3^*^	Sodium channel protein type 10 subunit alpha (Peripheral nerve sodium channel 3) (PN3) (Sensory neuron sodium channel) (Sodium channel protein type X subunit alpha) (Voltage-gated sodium channel subunit alpha Nav1.8)	Scn10a Sns
Q6PFX8	N-acetylaspartylglutamate synthase A (NAAG synthetase A) (NAAGS) (EC 6.3.2.41) (N-acetylaspartylglutamylglutamate synthase A) (EC 6.3.2.42) (Ribosomal protein S6 modification-like protein A)	Rimkla Fam80a Rimk
Q6NZK8	Protein tyrosine phosphatase domain-containing protein 1 (EC 3.1.3.-)	Ptpdc1
Q6A009	E3 ubiquitin-protein ligase listerin (EC 2.3.2.27) (RING finger protein 160) (RING-type E3 ubiquitin transferase listerin) (Zinc finger protein 294) (Zfp-294)	Ltn1 Kiaa0714 Lister Rnf160 Zfp294 Znf294
Q62147	Sarcospan (K-ras oncogene-associated protein) (Kirsten-Ras-associated protein)	Sspn Krag
Q61245	Collagen alpha-1(XI) chain (COBA1)	Col11a1
Q60632	COUP transcription factor 1 (COUP-TF1) (COUP transcription factor I) (COUP-TF I) (Nuclear receptor subfamily 2 group F member 1) (V-erbA-related protein 3) (EAR-3)	Nr2f1 Erbal3 Tfcoup1
Q5FW60	Major urinary protein 20 (Darcin) (Major urinary protein 24)	Mup20 Mup24
Q3V4B5	COMM domain-containing protein 6	Commd6
Q3V3R4	Integrin alpha-1 (CD49 antigen-like family member A) (Laminin and collagen receptor) (VLA-1) (CD antigen CD49a)	Itga1
Q3UZV7	UPF0577 protein KIAA1324-like homolog (Estrogen-induced gene 121-like protein) (EIG121L)	*NA*
Q3UR32^*^	P2X purinoceptor 3 (P2X3) (ATP receptor) (Purinergic receptor)	P2rx3
Q3UPR9	Somatomedin-B and thrombospondin type-1 domain-containing protein (RPE-spondin)	Sbspon Gm106 Rpesp
Q3TZX8	Polynucleotide 5′-hydroxyl-kinase NOL9 (EC 2.7.1.-) (Nucleolar protein 9)	Nol9
Q01339	Beta-2-glycoprotein 1 (APC inhibitor) (Activated protein C-binding protein) (Apolipoprotein H) (Apo-H) (Beta-2-glycoprotein I) (B2GPI) (Beta(2)GPI)	Apoh B2gp1
P82347	Delta-sarcoglycan (Delta-SG) (35 kDa dystrophin-associated glycoprotein) (35DAG)	Sgcd
P70271	PDZ and LIM domain protein 4 (LIM protein RIL) (Reversion-induced LIM protein)	Pdlim4 Ril
P70160^*^	Calcitonin	Calca Calc
P70158	Acid sphingomyelinase-like phosphodiesterase 3a (ASM-like phosphodiesterase 3a) (EC 3.1.4.-)	Smpdl3a Asml3a
P57724	Poly(rC)-binding protein 4 (Alpha-CP4)	Pcbp4
P51885	Lumican (Keratan sulfate proteoglycan lumican) (KSPG lumican)	Lum Lcn Ldc
P51642^*^	Ciliary neurotrophic factor (CNTF)	Cntf
P50608	Fibromodulin (FM) (Collagen-binding 59 kDa protein) (Keratan sulfate proteoglycan fibromodulin) (KSPG fibromodulin)	Fmod
P50285	Dimethylaniline monooxygenase [N-oxide-forming] 1 (EC 1.14.13.8) (Dimethylaniline oxidase 1) (Hepatic flavin-containing monooxygenase 1) (FMO 1)	Fmo1 Fmo-1
P48410	ATP-binding cassette sub-family D member 1 (Adrenoleukodystrophy protein) (ALDP)	Abcd1 Ald Aldgh
P47880	Insulin-like growth factor-binding protein 6 (IBP-6) (IGF-binding protein 6) (IGFBP-6)	Igfbp6 Igfbp-6
P46412	Glutathione peroxidase 3 (GPx-3) (GSHPx-3) (EC 1.11.1.9) (Plasma glutathione peroxidase) (GPx-P) (GSHPx-P)	Gpx3
P43135	COUP transcription factor 2 (COUP-TF2) (Apolipoprotein AI regulatory protein 1) (ARP-1) (COUP transcription factor II) (COUP-TF II) (Nuclear receptor subfamily 2 group F member 2)	Nr2f2 Aporp1 Arp-1 Arp1 Tfcoup2
P42925	Peroxisomal membrane protein 2 (22 kDa peroxisomal membrane protein)	Pxmp2 Pmp22
P40936	Indolethylamine N-methyltransferase (Indolamine N-methyltransferase) (EC 2.1.1.49) (EC 2.1.1.96) (Aromatic alkylamine N-methyltransferase) (Amine N-methyltransferase) (Arylamine N-methyltransferase) (Thioether S-methyltransferase) (TEMT)	Inmt Temt
P33622	Apolipoprotein C-III (Apo-CIII) (ApoC-III) (Apolipoprotein C3)	Apoc3
P24526	Myelin P2 protein (MYP2)	Pmp2
P21812	Mast cell protease 4 (mMCP-4) (EC 3.4.21.-) (MSMCP) (Myonase) (Serosal mast cell protease)	Mcpt4
P19258	Protein Mpv17 (Mpv-17)	Mpv17
P14483	H-2 class II histocompatibility antigen, A beta chain	H2-Ab1 H2-iabeta
P11589	Major urinary protein 2 (MUP 2)	Mup2
P08121	Collagen alpha-1(III) chain (CO3A1)	Col3a1
P08074	Carbonyl reductase [NADPH] 2 (EC 1.1.1.184) (Adipocyte protein P27) (AP27) (Lung carbonyl reductase) (LCR) (NADPH-dependent carbonyl reductase 2)	Cbr2
P06909	Complement factor H (Protein beta-1-H)	Cfh Hf1
P06684	Complement C5 (Hemolytic complement) [Cleaved into: Complement C5 beta chain; Complement C5 alpha chain; C5a anaphylatoxin; Complement C5 alpha' chain]	C5 Hc
P06330	Ig heavy chain V region AC38 205.12	*NA*
P04938	Major urinary protein 11	Mup11 Mup9
P03987	Ig gamma-3 chain C region	*NA*
P03975	IgE-binding protein	Iap
P03953	Complement factor D (EC 3.4.21.46) (28 kDa adipocyte protein) (Adipsin) (C3 convertase activator) (Properdin factor D)	Cfd Adn Df
P02762	Major urinary protein 6 (MUP 6) (Alpha-2U-globulin) (Group 1, BS6) (allergen Mus m 1)	Mup6
P01872	Ig mu chain C region	Ighm Igh-6
P01869	Ig gamma-1 chain C region, membrane-bound form	Ighg1 Igh-4
P01868	Ig gamma-1 chain C region secreted form	Ighg1 Igh-4
P01864	Ig gamma-2A chain C region secreted form (B allele)	*NA*
P01843	Ig lambda-1 chain C region	*NA*
P01837	Ig kappa chain C region	Igkc
O89013	Leptin receptor gene-related protein (Endospanin-1) (OB-R gene-related protein) (OB-RGRP)	Leprot Lepr Obr
O88630	Golgi SNAP receptor complex member 1 (28 kDa Golgi SNARE protein) (28 kDa cis-Golgi SNARE p28) (GOS-28)	Gosr1 Gs28
O88327	Alpha-catulin (Alpha-catenin-related protein) (ACRP) (Catenin alpha-like protein 1)	Ctnnal1 Catnal1
O70258	Epsilon-sarcoglycan (Epsilon-SG)	Sgce
O35744	Chitinase-like protein 3 (EC 3.2.1.52) (Beta-N-acetylhexosaminidase Ym1) (Chitinase-3-like protein 3) (ECF-L) (Eosinophil chemotactic cytokine) (Secreted protein Ym1)	Chil3 Chi3l3 Ym1
B1AVZ0	Uracil phosphoribosyltransferase homolog	Uprt
B1AS29	Glutamate receptor ionotropic, kainate 3 (GluK3) (Glutamate receptor 7) (GluR-7) (GluR7)	Grik3 Glur7
A2BIM8	Major urinary protein 18	Mup18 Mup16
Q9JK53	Prolargin (Proline-arginine-rich end leucine-rich repeat protein)	Prelp
Q9D4V3	Coiled-coil domain-containing protein 83	Ccdc83
Q91XI1	tRNA-dihydrouridine(47) synthase [NAD(P)(+)]-like (EC 1.3.1.-) (tRNA-dihydrouridine synthase 3-like)	Dus3l
P60060	Protein transport protein Sec61 subunit gamma	Sec61g
P27573^*^	Myelin protein P0 (MYP0) (Myelin peripheral protein) (MPP) (Myelin protein zero)	Mpz P0
P21183	Interleukin-5 receptor subunit alpha (IL-5 receptor subunit alpha) (IL-5R subunit alpha) (IL-5R-alpha) (IL-5RA) (CD antigen CD125)	Il5ra Il5r
Q8R502	Volume-regulated anion channel subunit LRRC8C (Factor for adipocyte differentiation 158) (Leucine-rich repeat-containing protein 8C)	Lrrc8c Ad158 Fad158
P11087	Collagen alpha-1(I) chain (Alpha-1 type I collagen) (CO1A1)	Col1a1 Cola1
P10493	Nidogen-1 (NID-1) (Entactin)	Nid1 Ent
P07758	Alpha-1-antitrypsin 1-1 (AAT) (Alpha-1 protease inhibitor 1) (Alpha-1-antiproteinase) (Serine protease inhibitor 1-1) (Serine protease inhibitor A1a) (Serpin A1a)	Serpina1a Dom1 Spi1-1
Q3TGW2	Endonuclease/exonuclease/phosphatase family domain-containing protein 1	Eepd1 Kiaa1706

Comparison of our PNS-enriched proteome with the content of the Human Protein Atlas (Uhlen et al., 2015; Thul et al., 2017). Non-matching proteins are listed. Given the previously shown good agreement between sensory neurons in mice and humans, these listed proteins are putatively enriched in the human PNS. Moreover, proteins reported by Ray and colleagues (Ray et al., 2018) to be DRG-enriched and conserved in humans are labeled with ^*^.

More importantly, our analysis highlighted many likely human PNS-enriched proteins whose functional relevance in the PNS is not well understood. Diverse collagens (COBA1, COEA1, CO1A1, CO3A1), along with their receptors (integrin alpha-1) and their binding proteins (fibromodulin) appear to be enriched in the PNS. Together with dermatopontin and lumican they are involved in GO_BP pathways of *extracellular matrix* and *collagen fibril organization*, respectively (enrichment FDR: 0.00038 and 0.00258). We also observed a significant enrichment of proteins implicated in GO_BP pathway of *lipid metabolic processes* (enrichment FDR: 0.037). These include diverse enzymes (e.g., branched-chain acyl-CoA oxidase, lysophospholipid acyltransferases 2 and 5, acid sphingomyelinase-like phosphodiesterase 3a and members of the myotubularin-related protein family). They catalyze lipid mediators with known roles in signaling cascades relevant for chronic pain such as sphingolipids (Patti et al., 2012) and lysophosphatidylcholine (LPC; Inoue et al., 2008), encouraging functional studies on these enzymes. Interestingly, our analysis also suggests several major urinary proteins (MUPs) to be enriched in the PNS. MUPs are known to bind lipophilic pheromones and are excreted in urine to mediate social communication in rodents (Hurst et al., 2001). However, their additional (patho) physiological functions are unclear and may be related to GO-BP *lipid storage* (enrichment FDR: 0.00731) and *glucose metabolism* (enrichment FDR: 0.037; Hui et al., 2009; Zhou et al., 2009). Moreover, the relationship between MUPs and neuropathic pain still needs to be determined.

Taken together, our PNS-enriched proteome will serve as a valuable resource for translational studies: This is encouraged by the high similarity between the mouse and human PNS (Sapio et al., 2016; Lopes et al., 2017; Ray et al., 2018) as well as our recent demonstration that mouse spectral libraries can be applied for protein profiling of human tissues (Bruderer et al., 2017b). Future work will require validated antibodies to reliably determine the localization of here identified PNS-enriched proteins in mouse and human. In particular, proteins which have not been characterized in the PNS before may represent novel players in peripheral pathologies, including pain, and should be queried for their functional role.

### Validation of region resolved proteomes in the SNI model of neuropathic pain

Thus far, we have demonstrated that PNS and SC proteomes can be comprehensively defined, and can also be employed to interrogate - at the protein level - the molecular set-up of tissues and pathologies across species. Next, we applied our DIA-MS analysis workflow to an ongoing endeavor in the field of chronic pain: The definition of a protein signature of chronic pain (Costigan, 2012). Knowledge of such a protein signature can be exploited for mechanism-oriented research and, ultimately, may offer opportunities for novel therapeutic approaches (Gereau et al., 2014; Baron et al., 2017). To this end we employed the widely-used SNI model, which mimics clinically relevant features including hypersensitivity to mechanical stimuli (tactile allodynia; Decosterd and Woolf, 2000). All SNI mice included in this study exhibited pronounced hypersensitivity to mechanical stimulation at 4 weeks, in contrast to Sham operated mice (Supplementary Figure 1).

We quantitatively compared our PNS and SC datasets obtained by DIA-MS from control (Sham surgery) mice with those which underwent SNI. We again had highest demands toward reproducibility and applied very stringent analysis criteria: proteins were only included in our analysis if their peptides were consistently detected in all six replicates, i.e., in three Sham and in three SNI replicates for a given tissue (4 mice/replicate, totaling 12 mice/condition; please see Methods for details). The heatmap of protein abundance across tissues and correlation analyses can be found in Supplementary Figure 4. Overall, more than 93% of proteins detected in control tissues were consistently quantified in all six replicates (complete list: Supplementary Table 8). These include 400 PNS-enriched proteins and 41 SC-enriched proteins. At this point it is unclear why the detection of SC-enriched proteins across all six replicates yielded only 55% (41/74) of proteins identified in control replicates described above (Supplementary Table 8). This contrasts with the high reproducibility of DIA-MS in PNS tissues and might hint at a massive downregulation of SC-enriched proteins below the detection limit in SNI samples.

**Figure 4:**
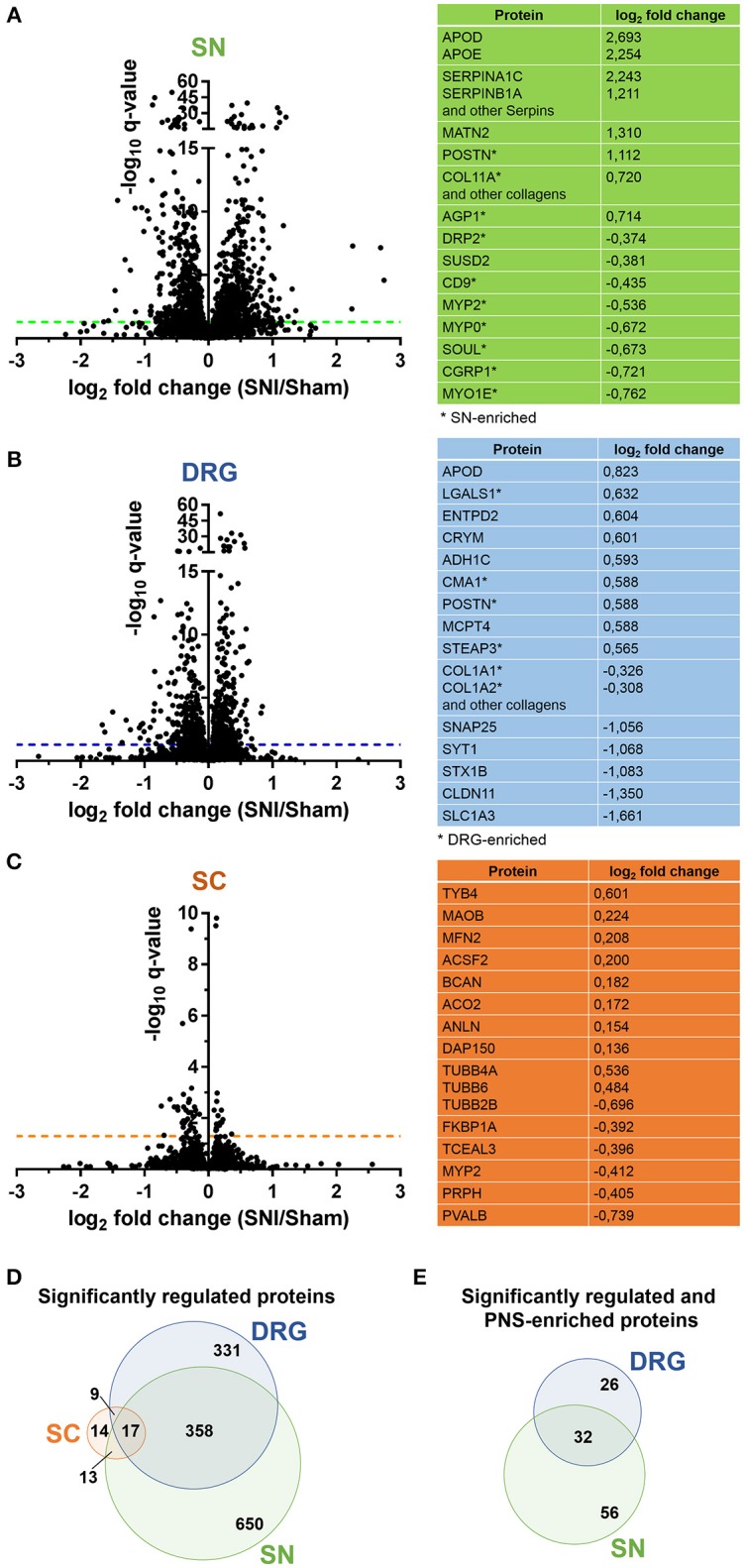
Proteome dynamics in the SNI model of neuropathic pain. **(A–C)** Volcano plots show the regulation of members of SN, DRG, and SC proteomes in Sham mice versus SNI mice. –log_10_ *q*-value is plotted against log_2_ fold change (SNI/Sham). The dotted horizontal line represents the cutoff for significance, *q* < 0.05. Tables list examples of regulated candidates in each region and respective log_2_ fold changes (complete list: Supplementary Table 8). **(D)** Venn diagram illustrates regulated (*q* < 0.05) proteins across SN, DRG, and SC proteomes. **(E)** Venn diagram illustrates regulated (*q* < 0.05) and differentially expressed proteins of the PNS-enriched proteome. In all panels the *q*-value represents the Benjamini-Hochberg-corrected *p*-value.

Upon statistical comparison of these data (please see Methods for details), we observed distinct and tissue-specific changes after SNI (Figures 4A–C; complete list: Supplementary Table 8). Most regulated proteins were in the SN (1026/2939, 34%) followed by DRG (715/3828, 18%) and SC (53/3584, 1.4%; Figures 4A–C). For the latter, more than half of the proteins were shared with the PNS as expected from aforementioned data (Figure 4D). Strikingly, 29% (114/400; 58 in DRG and 88 in SN; overlap in 32 proteins) of our PNS-enriched proteome showed significant alterations during neuropathic pain (Figure 4E). Among these, were 32 proteins that do not match the content of the Human Protein Atlas (Uhlen et al., 2015; Thul et al., 2017), thus, candidates considered to be putatively enriched in the human PNS (Table 2). These include several proteins involved in ECM integrity (e.g., some collagens, fibromodulin, and lumican).

**Table 2:** List of putative PNS-enriched candidate proteins in humans that are regulated upon SNI.

**Entry**	**Protein names**	**Gene names**	**Log**_**2**_ **(Pain/Sham)**		***q-*****value**	
			**DRG**	**SN**	**DRG**	**SN**
Q80X19	Collagen alpha-1(XIV) chain	Col14a1	0.28	0.11	0.000	0.001
P10493	Nidogen-1 (NID-1) (Entactin)	Nid1 Ent	0.17	0.08	0.000	0.049
Q9JK53	Prolargin (Proline-arginine-rich end leucine-rich repeat protein)	Prelp	0.39	−0.20	0.000	0.000
P51885	Lumican (Keratan sulfate proteoglycan lumican) (KSPG lumican)	Lum Lcn Ldc	0.41	−0.12	0.000	0.012
Q8CI59	Metalloreductase STEAP3 (EC 1.16.1.-) (Dudulin-2) (Protein nm1054) (Six-transmembrane epithelial antigen of prostate 3) (Tumor suppressor-activated pathway protein 6)	Steap3 Tsap6	0.56	0.84	0.000	0.000
Q01339	Beta-2-glycoprotein 1 (APC inhibitor) (Activated protein C-binding protein) (Apolipoprotein H) (Apo-H) (Beta-2-glycoprotein I) (B2GPI) (Beta(2)GPI)	Apoh B2gp1	0.35	0.14	0.000	0.265
P21812	Mast cell protease 4 (mMCP-4) (EC 3.4.21.-) (MSMCP) (Myonase) (Serosal mast cell protease)	Mcpt4	0.58	−0.27	0.001	0.013
P11087	Collagen alpha-1(I) chain (Alpha-1 type I collagen)	Col1a1 Cola1	-0.33	0.65	0.003	0.000
Q9ESB3	Histidine-rich glycoprotein (Histidine-proline-rich glycoprotein) (HPRG)	Hrg	0.23	0.11	0.011	0.280
P07758	Alpha-1-antitrypsin 1-1 (AAT) (Alpha-1 protease inhibitor 1) (Alpha-1-antiproteinase) (Serine protease inhibitor 1-1) (Serine protease inhibitor A1a) (Serpin A1a)	Serpina1a Dom1 Spi1-1	0.16	0.05	0.013	0.626
Q923B6	Metalloreductase STEAP4 (EC 1.16.1.-) (Dudulin-4) (Six-transmembrane epithelial antigen of prostate 4) (Tumor necrosis factor-alpha-induced adipose-related protein)	Steap4 Tiarp	0.42	NA	0.030	NA
Q3UPR9	Somatomedin-B and thrombospondin type-1 domain-containing protein (RPE-spondin)	Sbspon Gm106 Rpesp	0.17	0.03	0.043	0.717
P27573	Myelin protein P0 (Myelin peripheral protein) (MPP) (Myelin protein zero)	Mpz P0	0.42	−0.67	0.044	0.009
P46412	Glutathione peroxidase 3 (GPx-3) (GSHPx-3) (EC 1.11.1.9) (Plasma glutathione peroxidase) (GPx-P) (GSHPx-P)	Gpx3	0.37	0.00	0.049	0.945
P24526	Myelin P2 protein	Pmp2	0.25	−0.54	0.068	0.000
P01872	Ig mu chain C region	Ighm Igh-6	0.36	0.85	0.133	0.000
Q8R100	Protein FAM26E	Fam26e	NA	−0.37	NA	0.000
Q9ET38	Claudin-19	Cldn19	0.43	−0.30	0.359	0.001
P47880	Insulin-like growth factor-binding protein 6 (IBP-6) (IGF-binding protein 6) (IGFBP-6)	Igfbp6 Igfbp-6	NA	−0.34	NA	0.002
P06909	Complement factor H (Protein beta-1-H)	Cfh Hf1	0.20	0.47	0.172	0.004
P51642	Ciliary neurotrophic factor (CNTF)	Cntf	0.06	−0.25	0.681	0.005
P82347	Delta-sarcoglycan (Delta-SG) (35 kDa dystrophin-associated glycoprotein) (35DAG)	Sgcd	0.19	−0.22	0.168	0.009
P50608	Fibromodulin (FM) (Collagen-binding 59 kDa protein) (Keratan sulfate proteoglycan fibromodulin) (KSPG fibromodulin)	Fmod	0.59	−0.30	0.120	0.011
Q99JA0	Calcitonin gene-related peptide 1 (Alpha-type CGRP) (Calcitonin gene-related peptide I) (CGRP-I)	Calca Calc	0.02	−0.72	0.928	0.016
P70160	Calcitonin	Calca Calc	NA	−0.72	NA	0.016
Q8BH97	Reticulocalbin-3	Rcn3 D7Ertd671e	NA	0.21	NA	0.019
Q99LJ6	Glutathione peroxidase 7 (GPx-7) (GSHPx-7) (EC 1.11.1.9)	Gpx7	NA	0.78	NA	0.020
Q61245	Collagen alpha-1(XI) chain	Col11a1	-0.12	0.72	0.853	0.028
Q99JF5	Diphosphomevalonate decarboxylase (EC 4.1.1.33) (Mevalonate (diphospho)decarboxylase) (MDDase) (Mevalonate pyrophosphate decarboxylase)	Mvd	0.15	−0.23	0.629	0.030
Q9JJH1	Ribonuclease 4 (RNase 4) (EC 3.1.27.-)	Rnase4	NA	0.53	NA	0.037
P70271	PDZ and LIM domain protein 4 (LIM protein RIL) (Reversion-induced LIM protein)	Pdlim4 Ril	0.23	−0.28	0.383	0.048
Q6PFX8	N-acetylaspartylglutamate synthase A (NAAG synthetase A) (NAAGS) (EC 6.3.2.41) (N-acetylaspartylglutamylglutamate synthase A) (EC 6.3.2.42) (Ribosomal protein S6 modification-like protein A)	Rimkla Fam80a Rimk	-0.25	−1.64	0.298	0.049

*Graded color code: red, upregulated; green, downregulated; gray, unchanged. Up-and downregulation refer to proteins that were significantly regulated across all three replicates (Benjamini-Hochberg-corrected p-value, q < 0.05) upon SNI*.

Supporting our findings, a comparison with the catalog of protein-protein interactions involved in neuropathic pain (Jamieson et al., 2014), the PGD (Lacroix-Fralish et al., 2007), and the HPGDB (Meloto et al., 2017) revealed links of many SNI-regulated proteins to painful conditions in mouse and human. These include well-known pain-related gene products like nerve growth factor receptor (NGFR), annexin A2 (ANXA2), alpha2/delta subunit 1 of the voltage-dependent calcium channel (CACNA2d1), calcitonin gene-related peptide 1 (CGRP1 or CALCA), thrombospondin 4 (THBS4), and follistatin-like 1 (FSTL1; complete list: Supplementary Table 9).

More importantly, we found largely uncharacterized regulations. Several members of the aforementioned serpin family of serine protease inhibitors are upregulated in SN (e.g., SERPINA1C SERPINB6, SERPINC1, SERPINF1) or in DRG and SN (e.g., SERPINB1A, SERPING1, SERPINH1). GO-BP classification implicate these regulated SERPINs in *acute inflammatory responses*, in processes related to *wound healing* and, in general, related to the *regulation of stress responses*. Periostin (PSTN) is also prominently upregulated in SN and DRG in addition to being enriched in the PNS. There, it is likely secreted from dermal fibroblasts (Murota et al., 2017). It plays a role in cell adhesion and ECM integrity with a strong impact on inflammatory pathologies of the skin (e.g., atopic dermatitis) as well as the bowels (Koh et al., 2016; Murota et al., 2017).

Moreover, we have identified prominent protein downregulations after SNI. Dystrophin-related protein 2 (DRP-2) is among PNS-enriched proteins which are downregulated in SN. It is crucial for myelination in Schwann cells (Sherman et al., 2001) and, thus, has been linked to demyelinating neuropathies in both mouse and human (Sherman et al., 2001; Brennan et al., 2015). In the SC, a top-downregulated candidate is the low molecular weight protein thymosin beta4 (TYB4). Given its anti-inflammatory and angiogenic functions, TYB4 has consistently been shown to facilitate the repair and regeneration of diverse tissues and diabetic neuropathy mice (Philp and Kleinman, 2010; Wang et al., 2012). Hence, further exploration of TYB4 in the context of chronic pain may uncover an analgesic potential.

### Similarities and differences between inflammatory and neuropathic pain uncovered by retrospective analysis

Our results suggest a prominent regulation of candidate proteins with roles in inflammatory and immune processes upon SNI. This supports the notion that the immune system and inflammation are critical players underlying SNI pathologies (Krames, 2014; Ji et al., 2016; Sommer, 2016; Price and Gold, 2017; Cobos et al., 2018). Thus, we exploited the benefits of DIA-MS to retrospectively extend the analysis of our previously published datasets on chronic pain, specifically on the regulation of the DRG membrane proteome during inflammatory (upon injection of CFA) and neuropathic pain (SNI-model; Rouwette et al., 2016). As explained above (Figure 1) DIA-MS data are analyzed by means of spectral libraries. Hence, the depth and quality of the spectral library largely determine the proteome coverage one can obtain by a given experiment. The pan-mouse library generated here contains approximately 2-fold more proteins than our previously published spectral library (Rouwette et al., 2016). We employed this superior pan-mouse library to retrospectively extend our insights on pain-induced changes within the DRG membrane proteome.

Compared to our previous study (Rouwette et al., 2016), we identified dozens of additional proteins which appear to be regulated in only one of the two chronic pain models (CFA: 235, previously: 64; SNI: 156, previously 77). To give some examples, microsomal glutathione S-transferase 3 (MGST3) is downregulated during inflammatory pain. This protein belongs to a large family of peroxidase proteins involved in the production of leukotrienes and prostaglandin E (Bresell et al., 2005; Hayes et al., 2005). Notably, Hirai et al. (Hirai et al., 2017) have identified MGST3 through RNA sequencing after sciatic nerve entrapment (SNE) injury in rats, although it's role has not yet been examined in detail. Another example protein, Cancer susceptibility 4 (CASC4), is upregulated during inflammatory pain. As its name suggests, CASC4 is a proto-oncogene associated with breast and ovarian cancers (Anczuków and Krainer, 2015; Anczuków et al., 2015). It has yet to be actively discussed in the context of pain, but its transcript was found to be downregulated after spinal nerve transection (http://www.painnetworks.org:/ SNT_L5vsNAIVE_exonArrays). During neuropathic pain, galactin-1 (LGALS1) is upregulated. LGALS1 is a widely expressed lectin in a family of proteins implicated in inflammation, endometriosis, and cancer (Vergetaki et al., 2014). In the context of pain, it has been shown to mediate the severity of endometriosis (Vergetaki et al., 2014), as well as potentiate neuropathic pain in the dorsal horn (Imbe et al., 2003). Similarly, low-density lipoprotein receptor-related protein (LRP1) is found to be upregulated upon SNI. LRP1 is a large, multi-ligand signaling receptor which has been discussed in numerous contexts, ranging from cancer to oligodendritic cholesterol homeostasis to ECM remodeling (Etique et al., 2013; Lin et al., 2017). It has previously been considered as an “injury detection receptor” in addition to having a role in axonal regeneration after spinal injury (Yoon et al., 2013). The detection of LRP1 modulation after SNI in this retrospective analysis further hints at an important role after nerve injury.

Furthermore, we identified five times more proteins which are regulated in both pain models (55 proteins, previously: 11; Figure 5; complete list: Supplementary Table 10). While extensive work is needed to assess the functional relevance of these 55 shared proteins, their regulation in two etiologically different chronic pain models suggests a role as universal players in chronic pain. Here, we discuss some examples in more detail: One notable example is the alpha 2 isoform of Na+/K+ ATPase (ATP1A2). ATP1A2 is highly expressed in the brain, and has been implicated in cortical spreading depression and migraines (Unekawa et al., 2017). In fact, ATP1A2 represents one of the known susceptibility loci of familial hemiplegic migraine in humans (Friedrich et al., 2016). We also found many hits uncharacterized in the context of chronic pain which may warrant further study. Among them, we identified 2′,3′-cyclic-nucleotide 3'-phosphodiesterase (CNP), a myelin protein upregulated in both pain models, which is highly expressed in oligodendrocytes and Schwann cells (de Monasterio-Schrader et al., 2012). CNP is thought to play a role in RNA metabolism (Gravel et al., 2009) and has also been linked to neuroinflammation in Alzheimer's disease, Down Syndrome, and schizophrenia (Barley et al., 2009). Recent work suggested that its role in axonal support is biased toward sensory axons in the PNS (http://hdl.handle.net/11858/00-1735-0000-0023-964D-8) with details remaining to be explored. Interestingly, the macrophage mannose receptor 1 (MMR or MRC1) appears to be differentially regulated across both pain models (CFA down- and SNI upregulated). MMR is a cell surface receptor involved in host immune responses (Schuette et al., 2016). It contributes to the suppressive state of macrophages (Schuette et al., 2016). As such, MMR is upregulated by interleukin 4 upon nerve injury, which correlates with the amelioration of behavioral hypersensitivity (Kiguchi et al., 2015). Its differential regulation in the CFA- and SNI-model likely reflects its immunomodulatory role in macrophages and deserves further investigations.

**Figure 5:**
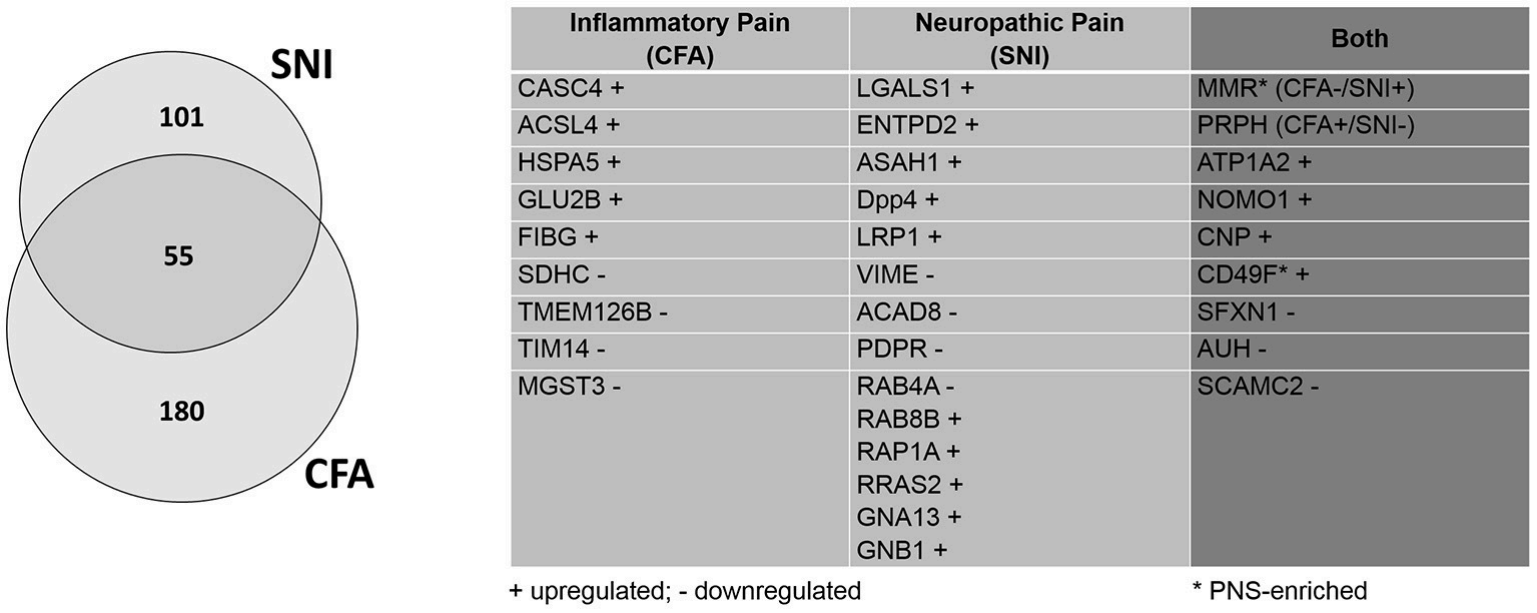
Retrospective analysis of DRG membrane proteome dynamics in two chronic pain models. Application of our pan-mouse library for retrospective analysis of our recently published datasets on mean abundance changes of DRG membrane proteins in the SNI-model of neuropathic pain and the CFA-model of inflammatory pain (Rouwette et al., 2016). Venn diagram illustrates the proportions of significantly regulated (across all three replicates; Benjamini-Hochberg-corrected *p*-value, *q* < 0.05) DRG membrane proteins. The table lists examples of regulated (+, upregulated; -, downregulated) candidates across both pain models (complete list: Supplementary Table 10).

In contrast to our current study on whole DRG, our previous work was performed on membrane-enriched DRG samples (Rouwette et al., 2016). As such, this retrospective analysis is not exhaustive. Nevertheless, it is highly valuable for deciphering the regulation of protein levels at membranes compared to whole-tissue data (the latter reflecting the overall cellular abundance of proteins). To highlight an example, several G-protein signaling components (e.g., Ras-related proteins: RAB8B RAB4A, RAP1A, RRAS2; guanine nucleotide-binding protein subunit alpha-13, GNA13) display changes upon SNI in the DRG membrane proteome (Supplementary Table 10), which could not be detected in whole tissue lysates. This suggests that subcellular proteome analysis can provide additional and insightful knowledge about the regulation of proteins during pathological conditions.

From a technical point of view, we significantly increased the depth of our previous data using the pan-mouse library generated here without requiring new tissue samples. This provides a proof-of-principle to how DIA-MS can open new avenues toward standardizing and continuously improving proteome data in the pain community.

### A protein network point-of-view on neuropathic pain

Rarely do proteins exert their functions alone. Rather, they are embedded in protein complexes and larger protein networks which enable the regulation and plasticity of cellular pathways (Alberts, 1998; Rouwette et al., 2015). Knowledge of such protein networks may therefore contribute to a mechanistic understanding of pathophysiological processes underlying SNI. This is in line with emerging approaches in network medicine for modulating diseases (Chapman et al., 2008; Antunes-Martins et al., 2013; Borsook and Kalso, 2013). We thus inquired whether significantly regulated proteins upon SNI cluster in distinct networks and signaling pathways.

**Figure 6:**
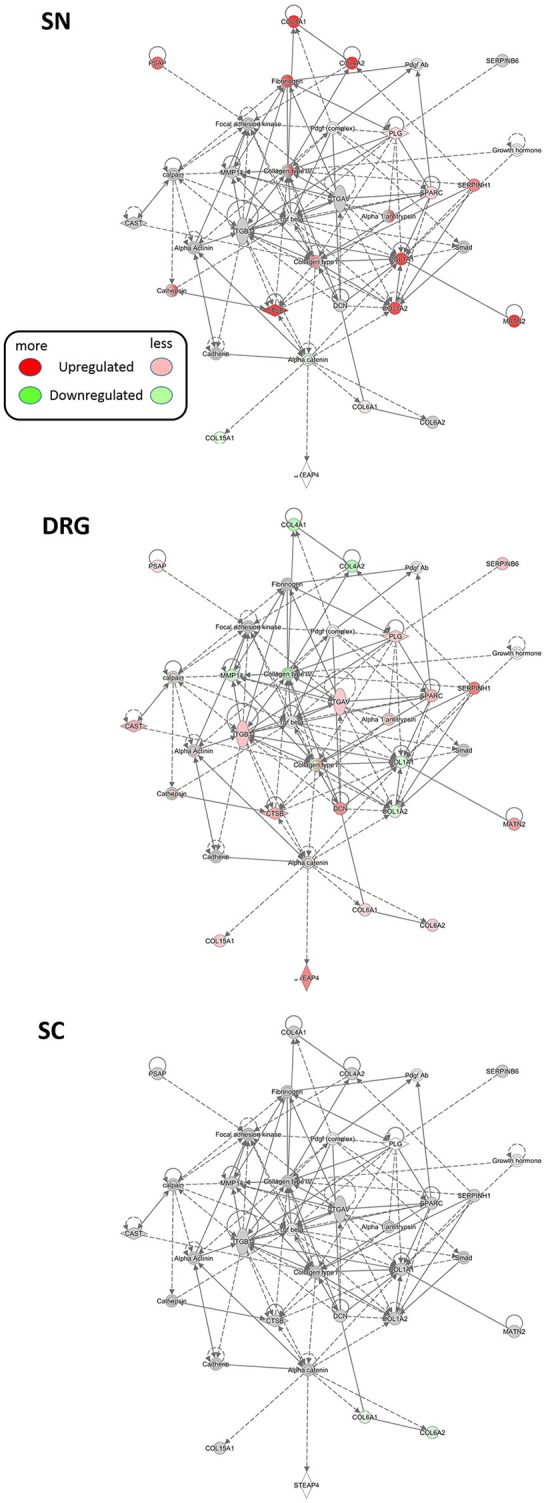
Region-resolved changes upon SNI in a protein network implicated in dermatological and inflammatory diseases (Network #20, Supplementary Table 11). Differential regulation of mean protein abundance across SN, DRG, and SC becomes apparent and is visualized by a graded color code. Node color code: red, upregulated; green, downregulated; gray, unchanged; white, not detected. Predicted relationship among nodes is indicated by dashed lines. All graphs are created by IPA®. Up-and downregulation refer to proteins that were significantly regulated across all three replicates (Benjamini-Hochberg-corrected *p*-value, *q* < 0.05) upon SNI.

Using IPA® we first broadly determined the interactions of regulated proteins within known, experimentally verified networks across all three examined tissues (Krämer A et al., 2014). Membership of regulated proteins in these networks is scored based on the in-built Fisher's exact test by IPA® (Supplementary Tables 11–13, which list members of each network). Besides shared networks, we detected prominent regional specificity of protein regulation within these networks. For example, several collagens and integrins implicated in ECM integrity are co-clustered with others in a network relevant for dermatological and inflammatory diseases (Network #20, Supplementary Table 11). Network analysis highlights their regulation upon SNI in a region-specific manner (Figure 6): strong upregulation of COL1A1, COL1A2, COL4A1, and COL4A2 in SN contrasts their downregulation in DRG. They are unchanged in SC. These data lend further support to the implication of ECM signaling in PNS processes associated with SNI.

**Figure 7:**
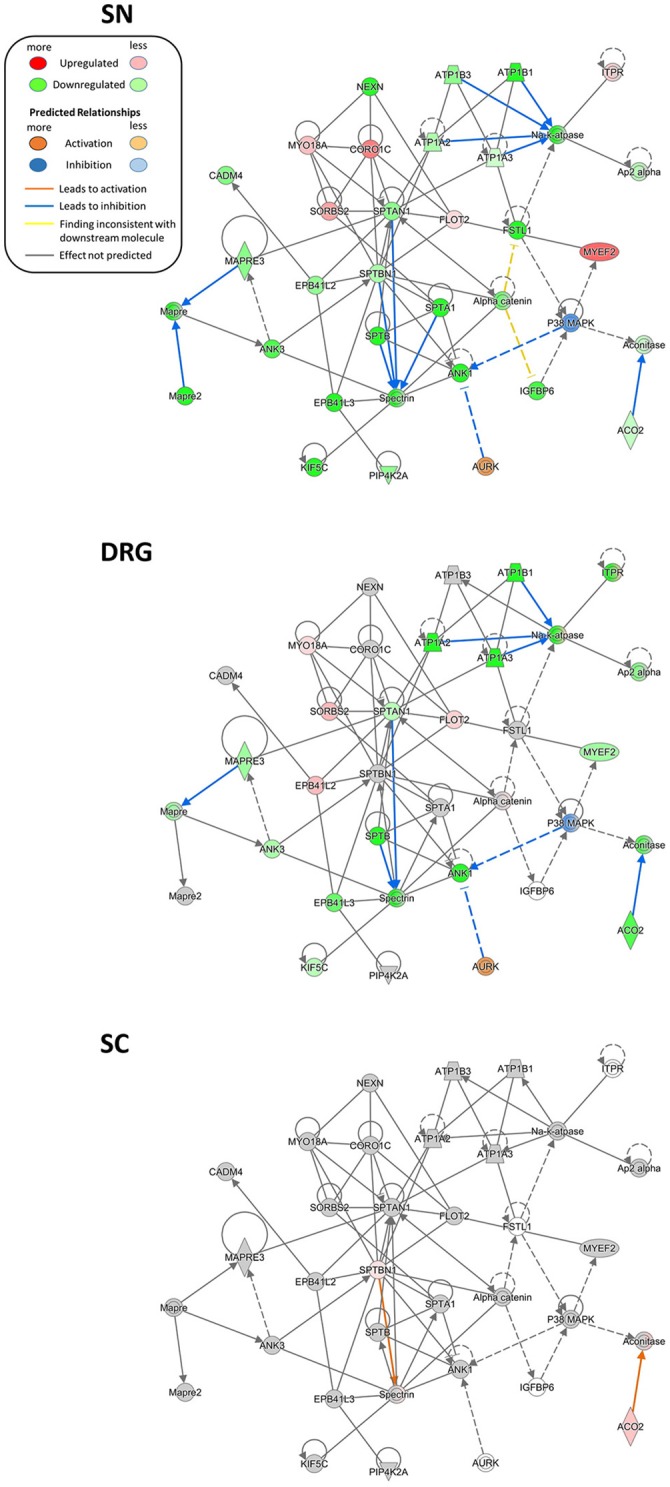
Region-resolved changes in the FSTL1-NKA-protein network upon SNI. Differential regulation of mean protein abundance across SN, DRG, and SC becomes apparent and is visualized by a graded color code. Node color code: red, upregulated; green, downregulated; gray, unchanged; white, not detected. Moreover, causal network analysis predicts differences in activation (line color orange) and inhibition (line color blue) of distinct connections between members of this network. Predicted relationship among nodes is indicated by dashed lines. All graphs are created by IPA®. Up-and downregulation refer to proteins that were significantly regulated across all three replicates (Benjamini-Hochberg-corrected *p*-value, *q* < 0.05) upon SNI.

We additionally critically assessed the possibility that our data can provide novel network-level insights into the pathology of proteins already described to be involved in pain. To this end, we turned to the regulation of several members of the sodium-potassium ATPase (NKA) family as an example. NKAs are significantly downregulated in our PNS dataset upon SNI. Two NKA members, ATP1A1A and ATP1B1, were reported to set sensory neuron excitability and to be controlled by follistatin-like protein 1 (FSTL1; Li et al., 2011b). Interestingly, a reduction of this FSTL1-NKA system in primary afferents correlated with neuropathic pain behaviors in rodents (Li et al., 2011a). In line with these findings, we not only observed an overall downregulation of the FSTL1-NKA system upon SNI, but could also visualize associated and partially modulated proteins within the same network in a region-dependent manner (Figure 7). These include the additional downregulation of several other NKA family members (A3, B3, and A2) in SN. Moreover, IPA® causal analysis (Krämer A et al., 2014) further suggests an interaction and inhibition of spectrin family members in the PNS compared to the SC.

**Figure 8:**
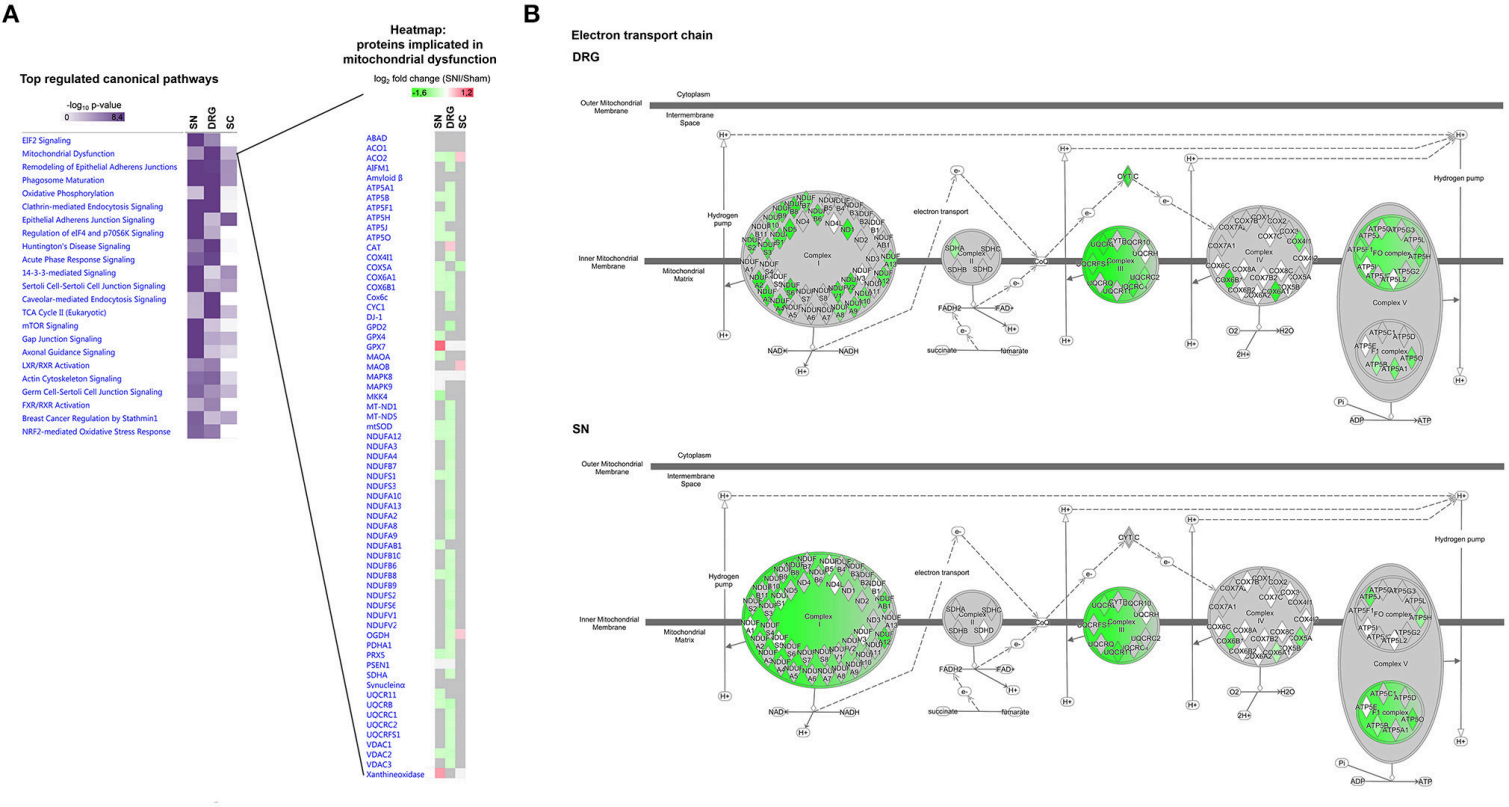
Region-resolved regulation of canonical pathways upon SNI. **(A)** *Left*, annotation matrix of pathway enrichment analysis for proteins regulated across all three replicates upon SNI in SN, DRG, and SC (scored based on the in-built Fisher's exact test by IPA® using the –log_10_ *p*-value). *Right*, heatmap depicts mean abundance changes (log_2_ fold change SNI/Sham; across all three replicates) of regulated proteins implicated in “*mitochondrial dysfunction.”* Color code: red, upregulated; green, downregulated; gray, unchanged; white, not detected. **(B)** Schematic representation and comparison of differentially regulated proteins in the electron transport chain (DRG versus SN). Mean protein abundance changes of significantly regulated proteins across all replicates are coded by a color gradient. Color code: red, upregulated; green, downregulated; gray, unchanged; white, not detected. All matrices and graphs are created by IPA®. Up-and downregulation refer to proteins that were significantly regulated across all three replicates (Benjamini-Hochberg-corrected *p*-value, *q* < 0.05) upon SNI.

We then went on to employ IPA® to interrogate regulated proteins associated with canonical cellular pathways. Overall, we detected changes in diverse pathways, many of which were differentially modulated in SN compared to DRG or SC (Figure 8A left). A notable example are interconnected pathways of *mitochondrial dysfunction, oxidative stress response, oxidative phosphorylation* and *TCA cycle*, known to underlie the diverse pathology of chronic pain (Baloh, 2008; Akude et al., 2011; Flatters, 2015). Figure 8A presents a heatmap comparing mean relative abundance changes of proteins involved in *mitochondrial dysfunction* across analyzed regions, with DRG being the most affected. These results are schematically visualized in Figure 8B.

Importantly, the prominent effect of SNI on mitochondrial proteins in DRG is in line with our retrospective analysis of the DRG membrane proteome. This was obtained from an independent cohort of mice in our previous study (Supplementary Table 10; Rouwette et al., 2016). Remarkably, thus, we found differences in the regulation of protein levels between data from DRG membranes compared to whole DRG. In DRG membrane samples we measured an upregulation of many proteins belonging to the mitochondrial respiratory chain (e.g., NDUFV1, NDUFS4, NDUFS5, NDUFA5, and NDUFS3; Supplementary Table 10) in contrast to their downregulation in whole DRG (Supplementary Table 8). These data may provide valuable insights into subcompartmental regulation, particularly regarding mitochondria. Ninety-nine percent of mitochondrial proteins are encoded by nuclear genes, thus their synthesis at cytosolic ribosomes, their cytosolic abundance, and their subsequent import into mitochondria are crucial for organelle function (Schmidt et al., 2010; Wiedemann and Pfanner, 2017). Protein import occurs in response to a variety of conditions, including oxidative stress, and is considered to contribute to mitochondrial dysfunction associated with diverse human diseases (MacKenzie and Payne, 2007). Currently, we can only speculate as to how here detected abundance changes may affect mitochondrial dysfunction. Nonetheless, our data build on previous work to suggest defects in mitochondrial import and membrane assembly play a role in neuropathic pain.

## Discussion

To obtain a thorough understanding of chronic pain, system wide mapping of the pain axis at the protein level is necessary. This has not yet been achieved. This is largely due to the fact that the technology required to reproducibly and quantitatively compare proteomes has emerged only recently. Here, we built upon these technical advances and offer a comprehensive protein compendium of three regions at the beginning of the pain neuraxis—the SN, DRG, and SC. Our results yield unprecedented insights into the proteome composition of these regions and their dynamics during neuropathic pain. We further provide a thus far unique dataset on proteins and protein networks that are highly enriched in the PNS and SC. Consequently, our study fills an eminent gap in the pain field and serves as a stepping stone for defining protein disease signatures and novel therapeutic targets of chronic pain.

Chronic pain is an extremely complex disease involving a myriad of molecular changes (Costigan et al., 2009; Patapoutian et al., 2009; Sommer, 2016; Price and Gold, 2017). This is likely why treatments interfering with single proteins exhibit only limited efficacy. Emerging medical research initiatives aim for a “mechanism-based” approach toward pain therapy (Borsook and Kalso, 2013; Gilron et al., 2013; Treede et al., 2013; Sommer, 2016; Vardeh et al., 2016; Baron et al., 2017). A prerequisite for this is a thorough, systems biology understanding of the complex and dynamic molecular interplay contributing to chronic pain (Chapman et al., 2008; Antunes-Martins et al., 2013; Borsook and Kalso, 2013).

Extensive genomic (Diatchenko et al., 2005, 2007, 2013; Sorge et al., 2012; Zorina-Lichtenwalter et al., 2016), epigenomic (Doehring et al., 2011; Denk and McMahon, 2012; Niederberger, 2014), and transcriptomic studies (Grace et al., 2012; Simonetti et al., 2013; Jeong et al., 2016) have provided highly valuable insights into the changes associated with painful pathologies in rodents and patients. Nonetheless, their predictability of phenotypes is limited (Mogil, 2012; Geyer et al., 2016; Liu et al., 2016; Sommer, 2016). This is especially the case for transient and/or dynamic biological processes, given the existence of various cellular buffering mechanisms (Liu et al., 2016). In contrast, the proteome constitutes a highly valuable source of molecular information for monitoring phenotypes - including disease onset and progression (Chen et al., 2015; Ebhardt et al., 2015; Sajic et al., 2015; Gazerani and Vinterhoj, 2016; Geyer et al., 2016, 2017). This advantage of standardized proteome analysis is extensively exploited in oncological research to define robust molecular tumor signatures (Hüttenhain et al., 2012; Cerciello et al., 2013; Takadate et al., 2013). Thus far, similar system-wide studies are lacking in the context of pain. In the pain field, most work published relies on gel-based approaches (typically 2D-PAGE) followed by data-dependent acquisition mass spectrometry (DDA-MS), often referred to as shotgun proteomics (Domon and Aebersold, 2010; Michalski et al., 2011). These methods have laid the foundation for proteomic studies of chronic pain (Huang et al., 2008; Olausson et al., 2012, 2015; Vacca et al., 2014). They are also known for their limitations regarding biased sampling, protein losses, and low reproducibility between studies (Domon and Aebersold, 2010; Michalski et al., 2011). Emerging data-independent acquisition mass spectrometry (DIA-MS; Venable et al., 2004; Panchaud et al., 2009; Carvalho et al., 2010) helps to overcome some of these limitations. In essence, DIA-MS enables the identification and quantification of proteins on a large scale and in a standardized and reproducible manner (Domon and Aebersold, 2010; Sajic et al., 2015). Therefore, it is ideally suited to interrogate pathology-related proteome changes (Hüttenhain et al., 2012; Cerciello et al., 2013).

Using DIA-MS, our previous study on the membrane proteome of DRG provided a glimpse of differential and pain-model specific changes in distinct cellular protein networks (Rouwette et al., 2016). Here, we extended our previous study and monitored global proteome changes in the SN, DRG, and SC. A wealth of previous work has shown that the PNS is (i) a significant driver of chronic pain, and (ii) offers higher therapeutic accessibility than brain areas implicated in pain (Puljak et al., 2009; Liem et al., 2013; Krames, 2014; Berta et al., 2017b). Consequently, recent therapeutic strategies aim to selectively target peripheral sensory neurons for pain relief (Sapunar et al., 2012; Liem et al., 2013; Haroutounian et al., 2014; Krames, 2014; Berta et al., 2017b). Despite its apparent clinical importance, the protein composition of the PNS has not been thoroughly investigated. Our study fills this gap and defines for the first time a set of PNS-enriched proteins in reference to two independent but highly similar and large-scale datasets of the mouse brain (our own results and results of others; Sharma et al., 2015). In addition, we cross-correlated these data with the Human Protein Atlas harboring protein information on more than 30 human tissues. This enabled us to define PNS-enriched protein signatures with high utility for translational research.

Strikingly, 32 of these putative PNS-enriched candidate proteins in humans displayed significant regulation in the SNI-model (Table 2). Among these, several are implicated in ECM integrity. The ECM is crucial for signal transduction and communication between extra- and intracellular spaces (Manninen and Varjosalo, 2017). Additionally, it is modulated in response to diverse stimuli. The ECM has been implicated in tissue homeostasis, as well as pathological processes triggered by nerve injury in the SC (Bartus et al., 2014; Tajerian and Clark, 2015). In chronic pain conditions, there is accumulating evidence for the role of ECM changes in hypersensitivity and long lasting maladaptation, particularly by affecting synaptic plasticity in the CNS (Tajerian and Clark, 2015). Yet, details about the role of its protein constituents for PNS (patho-) physiology remain to be investigated. Here, we reveal pronounced abundance changes of distinct proteins implicated in ECM integrity in our DRG and SN datasets. Collectively, these findings strongly suggest the involvement of ECM signaling in processes associated with SNI, and may warrant detailed studies on here identified regulated candidates.

Another notable feature of observed SNI-induced dynamics within the PNS is the high representation of proteins involved in immune signaling and inflammation (Figure 4 and Supplementary Table 8). This is in line with the evolving notion that DRG and SN harbor diverse immune cells (e.g., macrophages and T cells) with critical functions for normal physiology and during chronic pain (Krames, 2014; Ji et al., 2016; Sommer, 2016; Price and Gold, 2017; Cobos et al., 2018). In fact, a recent study demonstrated mechanistic differences in the contribution of immune cells to SNI-induced phenotypes: While they are largely dispensable for cold allodynia, immune cells appear to be critical for the development of tactile hypersensitivity likely via their interplay with sensory neurons (Cobos et al., 2018). Interestingly, our data reveal a significant overrepresentation of proteins associated with IPA® pathways of *acute phase response signaling* and *chronic inflammatory disorders* in PNS-enriched proteomes (Supplementary Table 3). Many of these are regulated upon SNI (e.g., diverse SERPINS; Figure 4 and Supplementary Table 8) and associated with other regulated proteins in distinct protein networks (Figure 6 and Supplementary Tables 11–13). Moreover, our retrospective analysis finds similarities in protein regulation during neuropathic and inflammatory pain (e.g., CNP, ATP1A2, NOMO1; Figure 5 and Supplementary Table 10). Hence, we uncover candidates for mechanistic follow-up studies on their role for immune and inflammatory signaling in the SNI-model.

Furthermore, we observe marked alterations in canonical pathways related to *mitochondrial dysfunction* (Figure 8). The importance of mitochondria in chronic pain is increasingly recognized (Baloh, 2008; Akude et al., 2011; Flatters, 2015). Our data extend existing knowledge by providing unprecedented and region-resolved details about alterations in specific mitochondrial proteins during chronic pain, particularly in the PNS. Interestingly, a recent transcriptome study showed that specifically injured neurons activate oxidative stress responses as early as 7 days after SNI (Berta et al., 2017a) suggesting that in our study a substantial part of observed changes may be derived from injured neurons.

In summary, we report here pronounced and differential dynamics upon SNI by looking at the proteome. On one hand, this knowledge will contribute to our understanding of diverse (patho-) physiological processes underlying the SNI-model (Decosterd and Woolf, 2000): not only chronic pain, but also tissue injury, activation of immune cells, and nerve de-/regeneration. On the other hand, our findings open opportunities for targeting regulated proteins and associated protein networks in treatments of chronic pain.

Of note, in order to reduce the number of mice and samples needed for this study in observation of the *3 Rs of animal research*, we used mice 28 days after Sham-surgery as controls to define tissue-resolved proteomes. While we cannot exclude proteome changes induced by Sham-surgery lasting for 28 days thereafter, several of our findings support the idea that our region-resolved proteomes largely reflect their “normal” protein content: (i) the here identified brain proteome is highly similar to previously published data in mice (Sharma et al., 2015), and (ii) the here identified PNS, SC proteomes, and region-enriched proteomes are in high accordance with legacy data on the molecular-set up of these tissues (Figure 3).

A common concern of tissue-based proteomics (and transcriptomics alike) experiments is the mixture of different cell types contained in each tissue. Therefore, the identified proteome alterations may be contributed by any cell type of the studied tissue. Further, changes of a distinct protein in one cell population may be masked by opposing changes in others. For example, our results in DRG likely reflect the proteome of diverse subpopulations of sensory neurons (Usoskin et al., 2015), immune cells, glia cells, and endothelial cells, many of which with prominent roles in painful pathologies (Krames, 2014; Ji et al., 2016; Sommer, 2016; Price and Gold, 2017). At this point we cannot judge the relative contributions of cell types to our DRG datasets; however, a comparison with existing transcriptome data of purified nociceptors (Thakur et al., 2014) and DRG subpopulations (Usoskin et al., 2015) enabled us to roughly estimate the proportion of neuronally expressed proteins to ~50%. These included known hallmark proteins of sensory DRG neurons, such as distinct ion channels and their interaction partners.

Another problem of tissue-based -omics arises from the intermingling of injured (dissected) and uninjured neurons in DRG and SN upon SNI. Moreover, fibers which were not primarily injured by the SNI surgery have been shown to be affected as well, exhibiting ectopic firing and contributing to the pathology (Costigan et al., 2009; Berta et al., 2017a). Recent transcriptome studies have elegantly compared injured with uninjured fibers (Hu et al., 2016; Berta et al., 2017a). Interestingly, it appears that such changes extend far beyond transcriptional regulation and need to be analyzed on the protein level, as well (Hu et al., 2016; Berta et al., 2017a).

Hence, future efforts should focus on efficiently labeling neuronal subpopulations *in vivo*. This would enable their separation followed by differential proteome analysis. Ideally, one would like to analyze single cell populations. So far, the analysis of single cell types is not amenable to comprehensive proteome profiling due to restrictions in sample amount and known limitations of profiling low abundant proteins in complex samples. Mass spectrometry workflows are rapidly evolving to improve both sensitivity and reproducibility. These advances involve novel acquisition strategies and computational analysis tools to maximize the amount of protein information obtained (Schubert et al., 2015; Tsou et al., 2015; Geyer et al., 2016; Bruderer et al., 2017a,b), all of which will open new possibilities in comprehensive proteome profiling of chronic pain.

Overall, we reveal dynamic changes of distinct protein networks during chronic pain (Figure 6 and Supplementary Tables 11–13; Figures 7, 8). This is highly relevant for state-of-the-art strategies in finding novel drug targets. As mentioned, therapeutic strategies targeting single proteins lack efficiency and often—depending on their organismal role—specificity to chronic pain (Borsook et al., 2014). The system-wide identification of critical hubs within defined protein networks, however, holds the promising potential to generate combinatorial therapeutic interventions, ideally less likely to cause severe side effects (Chapman et al., 2008; Barabási et al., 2011; Antunes-Martins et al., 2013; Borsook and Kalso, 2013; Chen et al., 2015). In order to promote a systems view on pain, Perkins and colleagues implemented “PainNetworks” (Perkins et al., 2013), Jamieson and colleagues summarized the “pain interactome” (Jamieson et al., 2014) and recent studies systematically mapped the DRG/PNS-enriched transcriptome by contrasting it to body-wide gene expression (Sapio et al., 2016; Ray et al., 2018). We now complement these efforts and provide a freely-available and searchable database (painproteome.em.mpg.de) harboring all data described in this work, i.e., region-resolved proteomes as well as SNI-associated proteome changes. In addition to encouraging future experimental investigations, these data may be used to test the application of designated network theory algorithms to find disease modules in large datasets (Zhang et al., 2009; Barabási et al., 2011; Shi et al., 2015; Kitsak et al., 2016). Examples of network theory impact include insightful work on cardiovascular diseases, cancer, and diabetes summarized elsewhere (Oti et al., 2006; Barabási et al., 2011; Zhou et al., 2014; Kitsak et al., 2016).

In conclusion, we have shown that the analysis of proteins and protein networks can promote novel insights into disease features of chronic pain. This may facilitate the development of mechanism-based therapies, which are urgently required for chronic pain syndromes (Gereau et al., 2014; Baron et al., 2017). We are confident that the pain community will quickly integrate such a protein-centric and systems-inspired view. To expedite this process, we comprehensively characterized the proteome and its pathology-associated dynamics along the beginning of the pain axis. Our results provide the necessary foundation for future studies examining the protein signature of chronic pain syndromes in different regions and in time.

## Data availability statements

All relevant data are contained within the manuscript: All datasets (generated and analyzed ones) for this study are included in the manuscript and the Supplementary Files. Moreover, all data is freely available in the searchable online database painproteome.em.mpg.de. Raw DIA-MS data was deposited in the PeptideAtlas repository (http://www.peptideatlas.org/).

## Author contributions

AMB performed the comparative and bioinformatics analysis of data and helped write the manuscript. JRS performed behavior experiments and biochemistry and helped write the manuscript. J-HS developed the database with the help of JRS. DG-V conceived the project and supervised the analysis of data. MS conceived, designed, and guided all parts of the project, and wrote the manuscript.

### Conflict of interest statement

MS received travel support by the German Pain Society (DGSS), which was sponsored by Astellas Pharma GmbH (Germany). DG-V received research support from Biognosys AG (Zurich, Switzerland). The remaining authors declare that the research was conducted in the absence of any commercial or financial relationships that could be construed as a potential conflict of interest.

## Supplementary material

Supplementary Figure 1 Quantification of unilateral (ipsilateral, ipsi) mechanical allodynia induced by SNI. The graph depicts average withdrawal latencies to innocuous mechanical stimulation of ipsi- and contralateral (contra, untreated control) paws before (pre) and 4 weeks (4 wk) after Sham and SNI-surgery, respectively. Mechanical allodynia is only observed 4 weeks after surgery in SNI-treated animals as indicated by a reduction of the withdrawal thresholds of ipsi paws compared to contra paws (*N* = 12 animals per condition). One-way ANOVA followed by Holm-Sidak's multiple comparison test, ^****^p < 0.0001 of corresponding ipsi vs. contra paws of SNI-treated animals.

Supplementary Figure 2 Regression analysis across Sham sample replicates. Sham replicates (control, C1-C3) are plotted per tissue, with scatter plots and Pearson's correlation coefficient shown per interaction. Protein intensity distributions (per replicate) are shown on the diagonal as kernel density estimates.

Supplementary Figure 3 Representation of gene products (top 50) of indicated sensory neuron subpopulations published by Usoskin et al. (2015) in our DRG proteome of Sham mice.

Supplementary Figure 4 DIA-MS: comparison of Sham with SNI samples. **(A)** Unsupervised clustering of z-scored normalized protein intensities from all replicates of SN, DRG, and SC tissue across SNI (pain, P1-P3) and Sham (control, C1-C3) replicates. The difference of replicate C2_DRG to other DRG samples is unexpected and likely explained by variability in sample preparation. Different colors of the y-axis dendrogram visualize exemplary clusters of proteins. **(B)** Regression analysis across SNI (pain, P1-P3) and Sham (control, C1-C3) replicates. Replicates are plotted per tissue (SN, DRG, and SC) with Pearson's correlation coefficient shown for each interaction.

Supplementary Table 1 The pan-mouse library.

Supplementary Table 2 DIA-MS results of proteins identified in tissues of Sham mice.

Supplementary Table 3 Pathway analysis in tissues of Sham mice.

Supplementary Table 4 List of proteins enriched in the PNS of Sham mice compared to brain areas.

Supplementary Table 5 List of proteins enriched in the SC of Sham mice compared to brain areas.

Supplementary Table 6 Comparisons of published studies with our PNS proteome, PNS-enriched proteome, SC proteome, and SC-enriched proteome.

Supplementary Table 7 Comparisons of our data to PGD, HPGDB and conserved DRG-enriched transcripts.

Supplementary Table 8 Proteins significantly regulated upon SNI within the PNS proteome, PNS-enriched proteome, SC proteome, and SC-enriched proteome.

Supplementary Table 9 Comparisons of published studies with proteins significantly regulated upon SNI within the PNS proteome and the PNS-enriched proteome.

Supplementary Table 10 Retrospective analysis of data on the DRG membrane proteome previously published by our laboratory.

Supplementary Table 11 Top biological networks harboring DRG proteins significantly regulated upon SNI.

Supplementary Table 12 Top biological networks harboring SN proteins significantly regulated upon SNI.

Supplementary Table 13 Top biological networks harboring SC proteins significantly regulated upon SNI.
