# Supplementary figures and images for: Region-Resolved Quantitative Proteome Profiling Reveals Molecular Dynamics Associated With Chronic Pain in the PNS and Spinal Cord
Source: Front Mol Neurosci. 2018 Aug 14;11:259. doi: 10.3389/fnmol.2018.00259 (PMC6103001; doi:10.3389/fnmol.2018.00259)

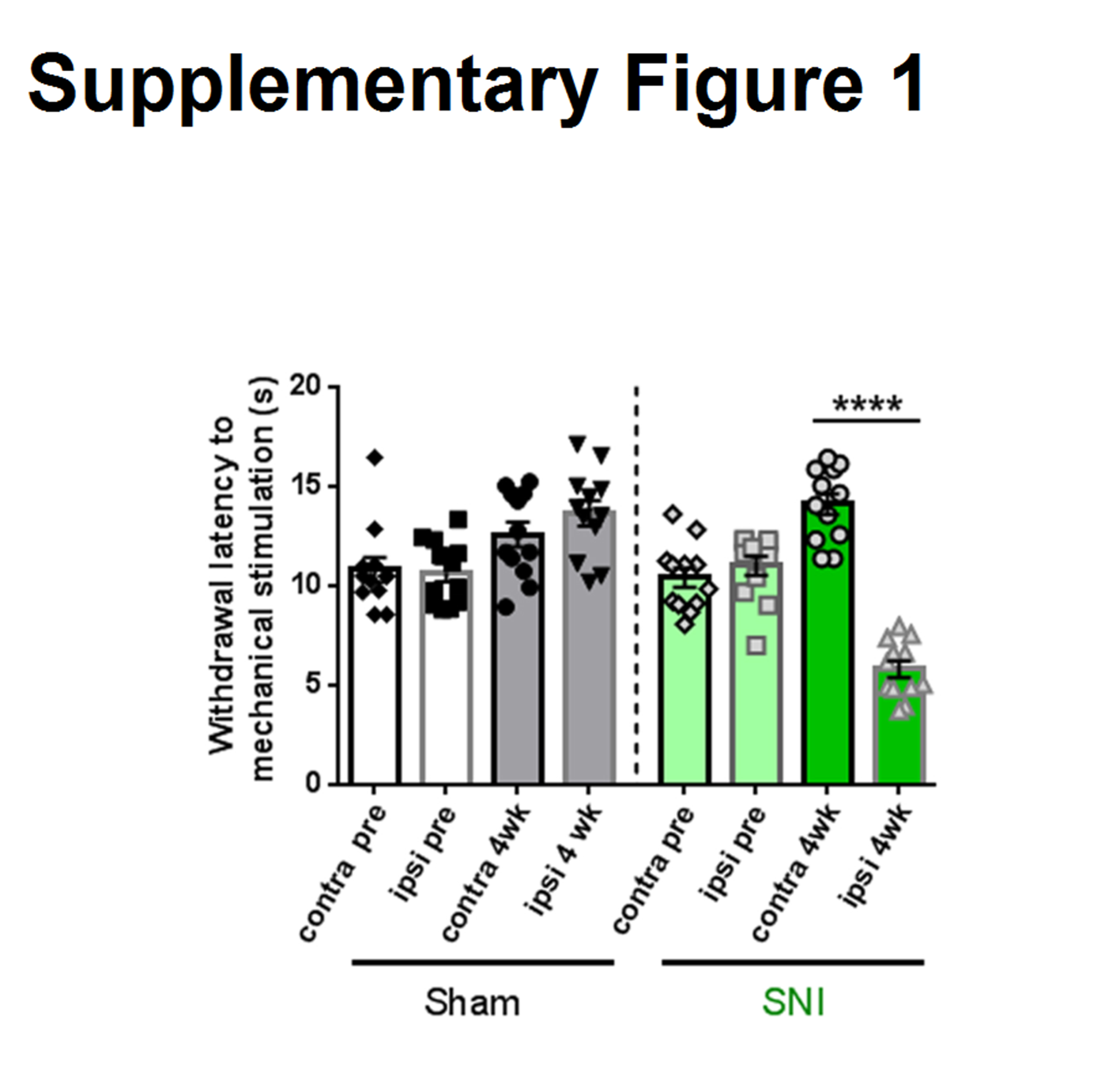

Supplement: Supplementary Figure 1 — Quantification of unilateral (ipsilateral, ipsi) mechanical allodynia induced by SNI. The graph depicts average withdrawal latencies to innocuous mechanical stimulation of ipsi- and contralateral (contra, untreated control) paws before (pre) and 4 weeks (4 wk) after Sham and SNI-surgery, respectively. Mechanical allodynia is only observed 4 weeks after surgery in SNI-treated animals as indicated by a reduction of the withdrawal thresholds of ipsi paws compared to contra paws (N = 12 animals per condition). One-way ANOVA followed by Holm-Sidak's multiple comparison test, ****p < 0.0001 of corresponding ipsi vs. contra paws of SNI-treated animals. [file Image_1.TIF]

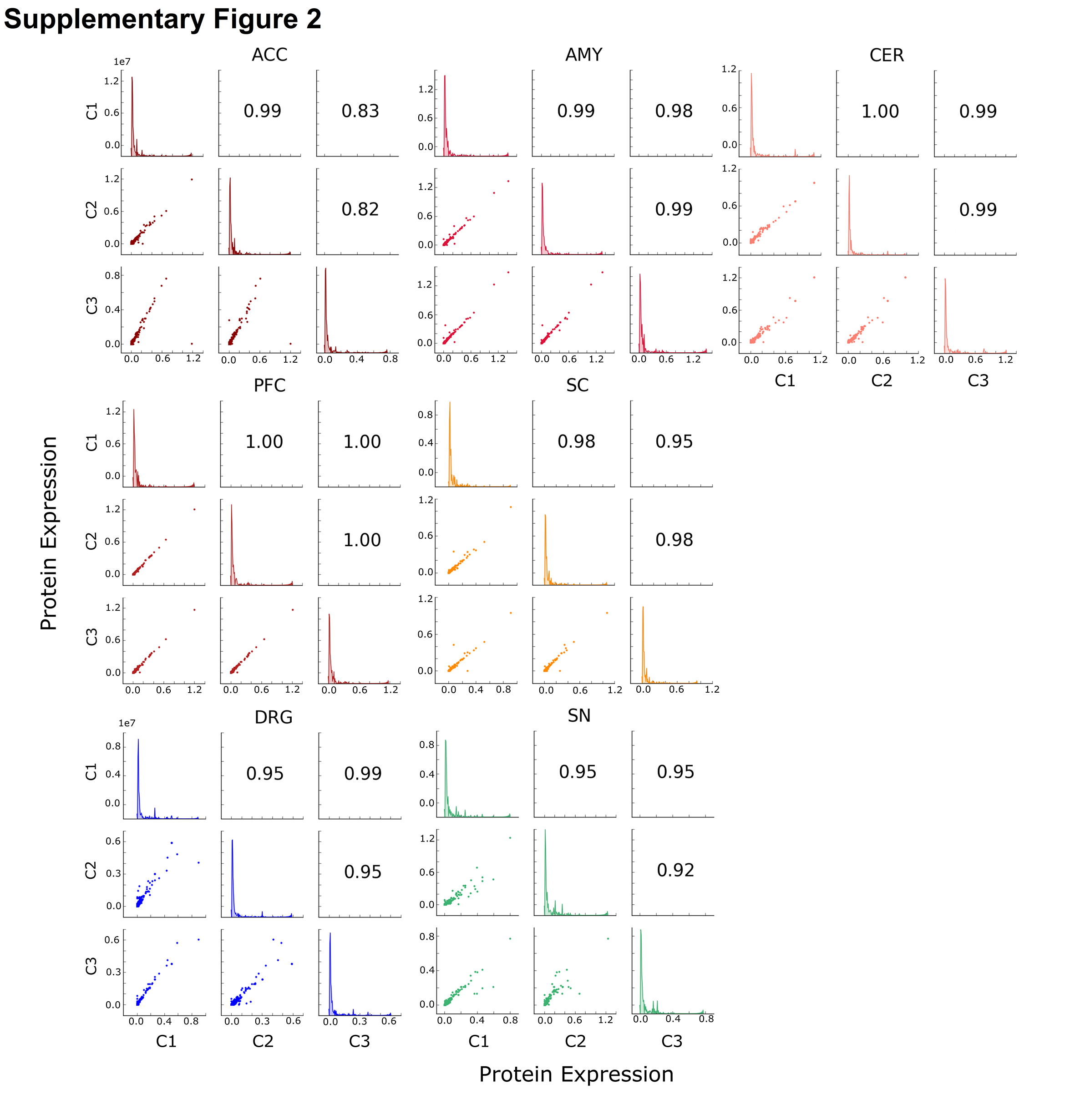

Supplement: Supplementary Figure 2 — Regression analysis across Sham sample replicates. Sham replicates (control, C1-C3) are plotted per tissue, with scatter plots and Pearson's correlation coefficient shown per interaction. Protein intensity distributions (per replicate) are shown on the diagonal as kernel density estimates. [file Image_2.TIF]

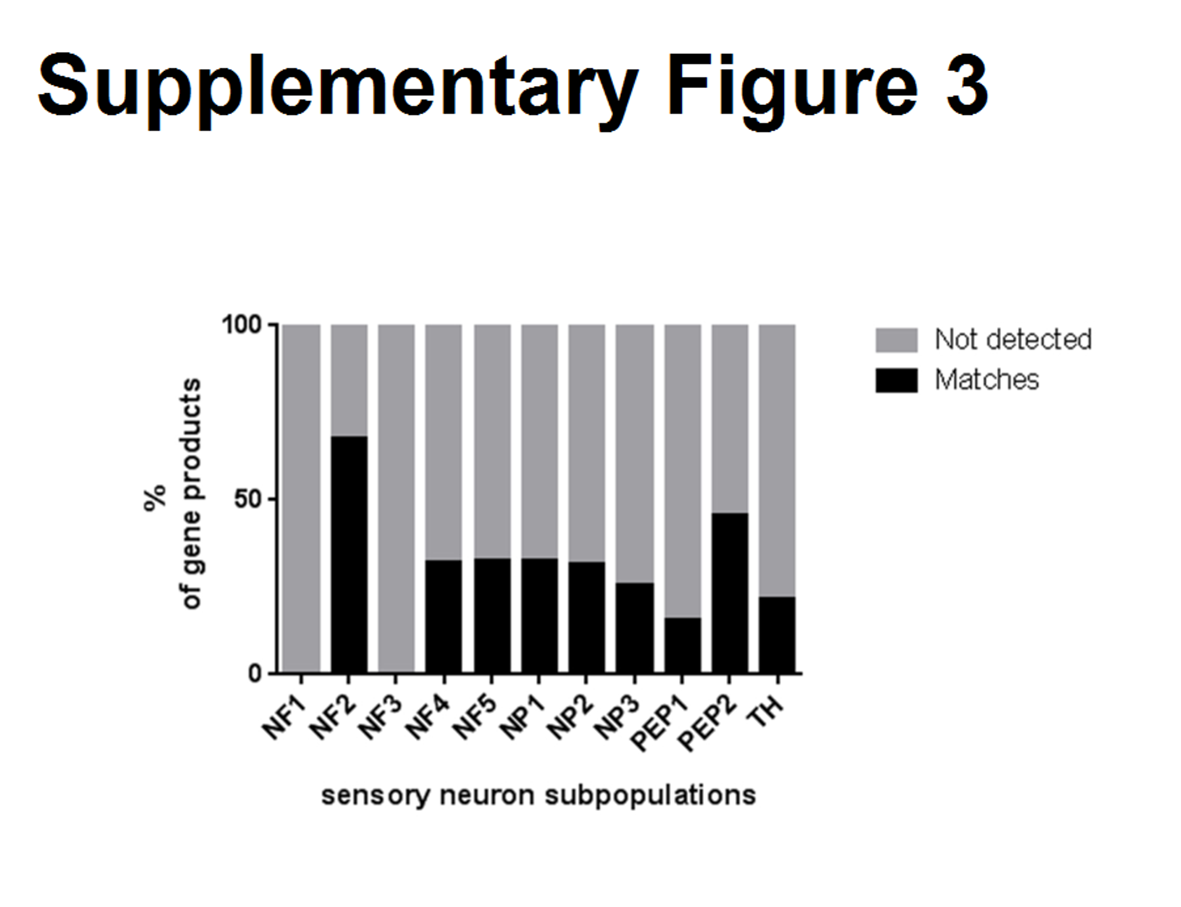

Supplement: Supplementary Figure 3 — Representation of gene products (top 50) of indicated sensory neuron subpopulations published by Usoskin et al. (2015) in our DRG proteome of Sham mice. [file Image_3.TIF]

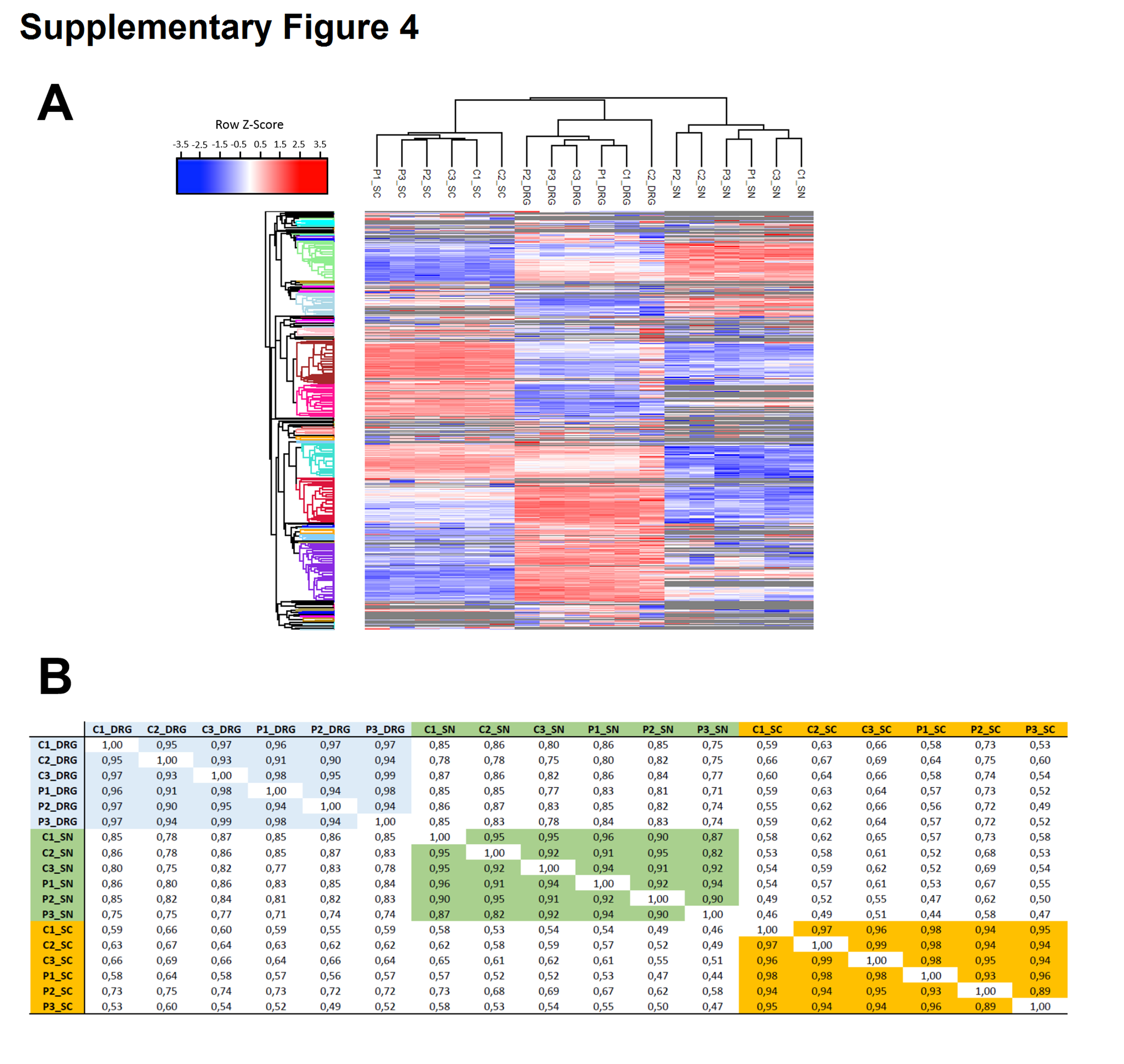

Supplement: Supplementary Figure 4 — DIA-MS: comparison of Sham with SNI samples. (A) Unsupervised clustering of z-scored normalized protein intensities from all replicates of SN, DRG, and SC tissue across SNI (pain, P1-P3) and Sham (control, C1-C3) replicates. The difference of replicate C2_DRG to other DRG samples is unexpected and likely explained by variability in sample preparation. Different colors of the y-axis dendrogram visualize exemplary clusters of proteins. (B) Regression analysis across SNI (pain, P1-P3) and Sham (control, C1-C3) replicates. Replicates are plotted per tissue (SN, DRG, and SC) with Pearson's correlation coefficient shown for each interaction. [file Image_4.TIF]
