# Supplementary material for: Region-Resolved Quantitative Proteome Profiling Reveals Molecular Dynamics Associated With Chronic Pain in the PNS and Spinal Cord
Source: Front Mol Neurosci. 2018 Aug 14;11:259. doi: 10.3389/fnmol.2018.00259 (PMC6103001; doi:10.3389/fnmol.2018.00259)
Supplement: Supplementary Table 11 — Top biological networks harboring DRG proteins significantly regulated upon SNI. [file Table_11.pdf]

Top biological networks harboring differentially regulated proteins in DRG

Network List

IPA Build version: 460209M

☐	ID	Molecules in Networl	Score	Focus Molecul	Top Diseases and Functions
☐	1	↑Acot1, ↑ACOT9, ↓ACOT13, acyl-CoA hydrolase, ↑ANKFY1, ↑APRT, ↑ARL2, ↓BCAT1, ↑CLIC1, ↑COL12A1, Collagen type V, ↑CPNE1, ↑DYSF, ↑EEF1B2, ↑EHD2, ↓EPB41, ↑ESYT1, ↑FN1, ↓GFM1, ↑HARS, ↓IDH3A, ↑ISLR, ↑KANK2, ↓LPCAT4, ↓NDUFS3, ↓PFAS, ↓PPP6R2, ↑SRM, ↑TAGLN2, ↑TARS, ↑TBCD, ↑TRIP12, ↑TXNDC17, UGT, ↑UGT1A6	48	32	Cell Morphology, Organ Morphology, Skeletal and Muscular Disorders
☐	2	60S ribosomal subunit, ↓CAND1, ↓CENPV, ↓CIRBP, ↓CRMP1, ↓CUL3, ↑EIF2S2, ↑ELAVL2, ↓FAM98B, ↑FTH1, hnRNP H, ↓HNRNPH1, ↓HNRNPU, ↓HP1BP3, ↑NDRG1, ↓PDXK, ↑PTBP1, ↓PTBP2, Ras homolog, ↓RBMX, Rnr, ↑RPL5, ↑RPL12, ↑RPL17, ↑RPL24, ↑RPLP0, ↑RPS8, ↑RPS11, ↑RPS16, ↑RPSA, ↑Rrbp1, ↓SDHA, ↑SND1, ↑UGGT1, ↑UPF1	45	31	RNA Post-Transcriptional Modification, Cancer, Cell Death and Survival
☐	3	↓COX6A1, ↓COX6B1, ↓Cox6c, ↓CYC1, Cytochrome bc1, cytochrome-c oxidase, ERK, ↓ETFA, ↓HSPE1, ↓ISCU, Mitochondrial complex 1, ↓MT-ND5, NADH dehydrogenase, ↓NDUFA2, ↓NDUFA3, ↓NDUFA4, ↓NDUFA8, ↓NDUFA9, ↓NDUFA10, ↓NDUFA12, ↓NDUFB6, ↓NDUFB7, ↓NDUFB8, ↓NDUFB9, ↓NDUFB10, ↓NDUFS2, ↓NDUFS6, ↓NDUFV1, ↓NDUFV2, ↓OPA1, ↓UQCRB, ↓UQCRC1, ↓UQCRC2, ↓UQCRFS1, ↓VDAC3	43	30	Developmental Disorder, Hereditary Disorder, Metabolic Disease
☐	4	↓ABLIM1, ↑ACTB, ↓AP1G1, ↓AP2M1, Arp2/3, ↑ARPC5, ↑ARPC1B, ↓CLTC, ↓CORO2B, ↑CSPG4, F Actin, ↑FLNA, ↑FLNB, ↑FLOT1, ↑FLOT2, Flotillin, ↓HPCAL1, ↑IQGAP1, ↑IQGAP2, ↑MYH9, ↓MYH10, ↑MYL6, ↑MYO18A, ↑MYO1C, ↓NCKIPSD, ↓OXR1, ↓PALM, Profilin, Rac, ↑SORBS1, ↑SORBS2, ↓SPTAN1, ↑SQRDL, ↑STOM, ↑VCL	43	30	Cell-To-Cell Signaling and Interaction, Cellular Assembly and Organization, Cellular Function and Maintenance
☐	5	↓ANK3, ↓Ap2b1, ↑CAPG, ↓CNTN1, ↓CNTNAP1, ↓CPLX1, Cytokeratin, Cytoplasmic Dynein, ↑DCTN1, ↑DCTN2, ↓DNM3, ↑DYNC1H1, ↓DYNC1I1, ↑Dync1i2, ↓FRY, ↓HSDL1, ↓KCNA1, ↓KCNA2, ↓KCNAB2, ↓KIF5C, Mapre, ↓MAPRE3, ↑MCAM, ↓NFASC, PI3K (complex), ↓RTN1, ↓RTN3, ↑RTN4, ↑SNTB1, ↑SNX1, ↑SNX2, ↑SNX5, ↓TAGLN3, tubulin (family), voltage-gated sodium channe	41	29	Cellular Assembly and Organization, Cellular Function and Maintenance, Nervous System Development and Function
☐	6	aldo, aminoacylase, ↑ASPA, ↓ATP6V0A1, ↓ATP6V0D1, ↓ATP6V1A, ↓ATP6V1B2, ↓ATP6V1C1, ↓ATP6V1D, ↓ATP6V1E1, ↓ATP6V1H, ↓CYFIP2, ↑DARS, ↓DMXL2, ↑EEF1D, ↑EPRS, ↓EPS15L1, ↓GRB2, H+-exporting ATPase, H+-transporting two-sector ATPase, ↓H1F0, ↑IDH2, ↓IDH3G, isocitrate dehydrogenase, ↑KARS, ↑LARS, ↓MCCC1, ↓NCKAP1, ↑PFN1, ↓RAB6B, ↑RARS, ↓SLC33A1, Vacuolar H+ ATPase, ↑WDR1, ↓WDR7	41	29	Cellular Function and Maintenance, Cellular Compromise, Molecular Transport
☐	7	↑ANPEP, ↑APOA1, ↑APOA4, ↑APOD, ↓CALB1, Ces, ↑Ces1b/Ces1c, ↓CLASP2, ↑CMA1, ↑CP, ↑CPA3, ↑DDAH2, ↑EPB41L2, ↓EPB41L3, ↑ESD, Ferritin, ↑FTL, ↑GPX3, hemoglobin, ↑HPX, ↓HSPA4L, ↑ME1, ↓MPP6, ↓NDRG2, Pdi, peroxidase (miscellaneous), ↑PRDX1, ↓PRDX5, ↑PRDX6, ↓PRUNE1, ↑RCC1, ↑STEAP3, ↑TF, Vegf, VLDL-cholestero	39	28	Free Radical Scavenging, Small Molecule Biochemistry, Endocrine System Disorders
☐	8	↑ANXA6, ↑ARCN1, Arf, ↑AVIL, ↑CALR, COP I, ↑COPA, ↑COPB1, ↑COPB2, ↑COPG1, ↑COPZ1, ↑EFEMP1, Gαq, ↑HSP90B1, ↑HYOU1, Lamin, Lamin b, ↓LMNB1, ↓LMNB2, mGluR, MHC Class I (complex), ↑PDIA3, ↑PDIA4, ↑PDIA6, ↑PITRM1, Pkc(s), ↑PLCD1, ↑PLEC, ↑PPIB, ↑PRKCSH, ↓SPTB, Tap, ↑TGFBI, ↑TXNDC5, ↑VWA1	34	26	Infectious Diseases, Cell Morphology, Cellular Development
☐	9	↑ACADL, ↓AIFM1, aldehyde dehydrogenase, aldehyde dehydrogenase (NAD), ALDH, ↓ALDH1A1, ↓ALDH1L1, ↓ALDH5A1, ↓ALDH6A1, ↑ALDH9A1, ↓ATP5B, ↑CAT, ↓COX4I1, ↑DBI, ↓DPP6, ↓GLUD1, glutathione peroxidase ↑GPX1, Insulin, Ldh (complex), ↑LRRC47, ↓MT-ND1, ↓MTCH2, ↓NDUFA13, ↓NDUFS1, ↓PHB, ↓PHB2, ↑PLIN3, Secretase gamma, ↑SELENBP1, ↓SLC25A12, Sod, ↓SOD2, ↓STOML2, Tnf (family)	34	26	Post-Translational Modification, Protein Synthesis, Protein Trafficking
☐	10	↑ARHGDIB, ↓CASKIN1, Dynamin, Endophilin, ↓ENO2, ↑F13A1, ↓GDI1, ↑GDI2, ↓MAG, ↑MAP4, ↑MICAL1, ↓NAPB, ↓NAPG, ↓NSF, ↓PLP1, Rab5, Rab11, ↓RAB3A, ↓RAB6A, Ras, Rho gdi, ↑SEPT2, ↓SEPT5, ↓SEPT6, ↓SEPT8, ↑SEPT9, Septin, ↓SH3GL2, ↓SNAP25, Snare, ↑SNCG, ↓STXBP1, Syntaxin, ↓SYT1, transglutaminase	32	25	Cellular Function and Maintenance, Molecular Transport, Cell-To-Cell Signaling and Interaction
☐	11	↓ACAD9, ↑ACLY, amylase, ATP synthase, ↓ATP5A1, ↓ATP5H, ↑CMPK2, ↑CNDP2, Collagen Alpha1, Eif2, Eotaxin, F1 ATPase, ↑FAM129A, ↓GPD1, ↓GPD2, JAK1/2, MIR124, ↑Mug1/Mug2 NDPK, ↑NME2, ↓PSIP1, Ribosomal 40s subunit, ↑RNH1, ↑SERPINB1, ↓SLC25A3, ↓SLC25A4, ↓SLC25A5, ↓SLC25A11, ↑STAT3, ↓SUCLA2, ↓SUCLG1, ↑TPD52, ↑TPD52L2, ↓TUFM, ↓VDAC2	32	25	Nucleic Acid Metabolism, Developmental Disorder, Hereditary Disorder
☐	12	↑ACAT2, acetyl-CoA C-acetyltransferase, adenosine-tetraphosphatase ↓AK1, Ant, ↑ANXA11, ↓ATP5F1, ↓AUH, ↓DLAT, ↓DLD, enoyl-CoA hydratase, ↑GAA, ↑GANAB, ↓GBAS, ↓GNG3, ↑HADHA, ↑HADHB, hexokinase, ↓HK1, HMG CoA synthase, ↑HMGCS2, Inflammasome (Nalp3, Asc, Casp1), Mapk, mediator, ↓OGDH, ↓PDHA1, ↓PDHX, ↓PEX5L, ↑PGD, ↓SAMM50, ↓SIRT2, ↓SORL1, Srebp, Vdac, ↓VDAC1	30	24	Developmental Disorder, Hereditary Disorder, Metabolic Disease
☐	13	BetaArrestin, ↓CKB, ↓CKMT1A/CKMT1B, Clathrin, ↑CLIC4, ↓Cox5b, ↑CRYAB, ↑CRYM, ↑DES, ↓DNAJA4, ↓DNAJC6, ↓DNAJC11, ENaC, ↓FKBP4, Gpcr, ↓GPHN, Hdac, ↓Hist1h1a, ↓Hnrnpa3, ↓HNRNPDL, ↓HPCA, HSP, Hsp70, Hsp90, Hsp22/Hsp40/Hsp90, ↓HSPA4, ↓HSPA9, ↓HSPH1, ↓MAPT, MHC Class II (complex), ↓NDRG4, ↑PRX, ↓STIP1, Tlr, ↓TUBA4A	30	24	Hereditary Disorder, Organismal Injury and Abnormalities, Skeletal and Muscular Disorders
☐	14	↓AAK1, ↓ABAT, adenosylhomocysteinase, ↓AGK, ↑AHCY, ↓AHCYL1, ↓AHCYL2, ↑ANXA5, Ap2 alpha, ↓ATP1A1, ↓ATP1A2, ↓ATP1A3, ↓ATP1B1, ↓ATP1B2, ↓ATP2A2, ↓ATP2B1, ↓ATP2B2, Ca2 ATPase, ERK1/2, G-Actin, ↓GOT1, ↑GPT2, Hepatic Transaminase, ITPR, ↓ITPR1, ↑ITPR3, ↑Ktn1, ↓MYO5A, Na+,K+-ATPase, Na-k-atpase, Ncx, Pkg, Pmca, ↑SH3BGRL, ↓SLC4A4	28	23	Molecular Transport, Cellular Function and Maintenance, Small Molecule Biochemistry
☐	15	↑ANXA2, ↑ANXA3, ↑ANXA7, ↓ARF6, Cdc2, CDK4/6, ↑COLGALT1, Cyclin A, Cyclin B, Cyclin D, Cyclin E, ↑DDOST, E2f, ↓ENO1, ↑FAM129B, ↑FHL1, ↑ITGB4, ↑LMNA, Mek, ↓NCDN, ↓NPEPPS, ↑OAT, p85 (pik3r), PDGF BB, ↑PRDX4, Rbp, ↑RBP1, ↑RPN1, ↑S100A4, ↓SCCPDH, Sos, ↑TALDO1, ↑VCP, ↑VIM, ↓YARS	28	23	Post-Translational Modification, Hereditary Disorder, Neurological Disease
☐	16	↓ACO2, ↑AKR1B1, ↓APP, ↓ATP5O, ↓BDH1, ↓CALB1, CCL2, ↓CEND1, ↓Cox6c, ↑CRYM, ↑DCLK1, ↑DPP3, ↑EGFR, ↓FAM213A, ↑Hrg, IL1B, ↓MAP6, methylamine, N-acetyl-L-aspartic acid, N-type Calcium Channel, ↓Ndufs5, NME6, peptidase, ↑PRRC1, PTGFR, ↓RAB18, ↑SERPINB1, ↓SLC1A3, ↓SLC25A25, ↓SNCA, ↓STX1B, ↓TMEM35A, ↑TPPP3, ↓VSNL1, ZXDC	28	23	Amino Acid Metabolism, Cell-To-Cell Signaling and Interaction, Molecular Transport
☐	17	Adaptor protein 1, ↓ANK1, AURK, ↑BCAM, ↓CAB39, Complement component 1, ↑CTNNA1, ↑DAG1, elastase, ↑ILK, Integrin alpha V beta 3, Jnk, ↑LAMA2, ↑LAMB1, ↑LAMB2, ↑LAMC1, Laminin, Laminin1, Laminin2, LIMS, ↑LIMS1, ↓MYO1D, ↑NID1, ↑NID2, ↑PARVA, ↑RHOC, ↓RHOT1, ↑RSU1, ↑SERPINF2, ↑SERPING1, ↑SLC4A1, Smad2/3, Stat3-Stat3, TMSB4, ↑TWF2	27	22	Cell Morphology, Organismal Injury and Abnormalities, Tissue Morphology
☐	18	↑ACAA1, acetyl-CoA C-acyltransferase, ↑AIMP1, Alpha actin, AP-3, ↓AP3B2, chymotrypsin, ↑CNN3, ↑COL18A1, Collagen type XVIII, ↓CS, ↑Cyb5r3, ↑ERAP1, Fascin, ↓FH, Gpd, ↑GSN, HLA-B27, ↑IARS, ↓INA, malate dehydrogenase, ↓MDH1, ↓MDH2, Myosin, NCOR-LXR-Oxysterol-RXR-9 cis RA, NFkB (complex), ↓PC, ↑PDLIM5, PPARα-RXRα, ↑PRPH, ↑Tpm1, ↑Tpm2, ↑TPM3, ↑Tpm4, Tropomyosin	25	21	Small Molecule Biochemistry, Cellular Function and Maintenance, Energy Production
☐	19	19S proteasome, 20s proteasome, 26s Proteasome, ↑ATOX1, ATPase, ↑DDX3X, Dishevelled, ↓ECI1, EIF4A, ↑EIF4A1, ↑EIF4A2, ↑EIF4E, EIF4F, Eif4g, ↑EIF4G1, ↑ENTPD2, ↑FSCN1, Gsk3, ↓LONP1, ↓Macf1, MAP1LC3, MHC CLASS I (family), nucleoside-triphosphatase P glycoprotein, ↑PABPC1, ↑PSMC3, PSMD, ↑PSMD2, ↑PSMD6, ↑PSMD7, ↑PSME1, ↑PSME2, ↓SFPQ, ↓TOM1L2, Ubiquitin	23	20	Protein Synthesis, RNA Post-Transcriptional Modification, Gene Expression
☐	20	Alpha 1 antitrypsin, Alpha Actinin, Alpha catenin, Cadherin, calpain, ↑CAST, Cathepsin, ↑COL15A1, ↓COL1A1, ↓COL1A2, ↓COL4A1, ↓COL4A2, ↑COL6A1, ↑COL6A2, Collagen type I, Collagen type IV, ↑CTSB, ↑DCN, Fibrinogen, Focal adhesion kinase, Growth hormone, ↑ITGAV, ↑ITGB1, ↑MATN2, ↓MMP14, Pdgf (complex), Pdgf Ab, ↑PLG, ↑PSAP, ↑SERPINB6, ↑SERPINH1, Smad, ↑SPARC, ↑STEAP4, Tgf beta	23	20	Dermatological Diseases and Conditions, Inflammatory Disease, Organismal Injury and Abnormalities
☐	21	Alpha tubulin, Beta Tubulin, ↑CALU, ↑CCT2, ↑CCT3, ↑CCT4, ↑CCT7, ↑CCT8, ↑CCT6A, ↓DNM2, Dynein, ↑EHD4, Erm, ↓Hist1h1e, Histone H1, Importin alpha, Importin beta, ↑IPO5, JAK, Karyopherin beta, ↑KPNB1, ↓MAP6, myosin-light-chain kinase, NCK, ↓NIPSNAP1, ↓PACSIN1, Pak, PI3K p85, ↑PPP2R2A, SRC (family), ↑STMN1, ↑TCP1, TEC/BTK/ITK/TXK/BMX, ↑TMEM43, ↑TNPO1	23	20	Cellular Assembly and Organization, Cell-To-Cell Signaling and Interaction, Reproductive System Development and Function
☐	22	alkylglycerophosphoethanolamine phosphodiesterase Angiotensin II receptor type 1, ↓BPHL, ↑CARM1, Cbp/p300, ↑EEF2, ↑FABP7, ↑FARSA, ↑FASN, ↓Gk, ↓GNA11, ↓GNAI1, ↓GNAO1, ↑IDH1, Lpa receptor, ↑MAPK3, N-cor, Nr1h, ↑NUCB1, Nuclear factor 1, PEPCK, ↓PFKM, PLA2, Plc beta, ↓PPP5C, Rar, ↑RPL7A, Rxr, SERCA, SWI-SNF, T3-TR-RXR, thymidine kinase, thyroid hormone receptor, ↑TKT, ↑VAT1	20	18	Cell Signaling, Molecular Transport, Nucleic Acid Metabolism
☐	23	AChR, Actin, ↓ALDOC, atypical protein kinase C, ↑CACNA2D1, Calmodulin, caspase, ↓CASQ1, ↓CNP, Cofilin, ↑DMD, ↓DNM1L, ↓DPYSL2, EGLN, ↓GDAP1, ↓GLUL, ↓LGI3, ↓MAP2, ↓Mff, Mlc, Mlcp, ↓MPI, ↓NCAM1, ↓Nefm, Nos, Pka catalytic subunit, PP1 protein complex group, PP2A, Ppp2c, ↑PTRF, Rb, Rock, ↑SERPINA3*, Spectrin, ↓VSNL1	20	18	Cell Morphology, Nervous System Development and Function, Tissue Morphology
☐	24	Adaptor protein 2, ↑AKR1A1, ↓ANK1, ↓ATP2B2, BCL2, BOK, BRCA1, C22orf29, CACNA1A, ↓CACNB4, ↑CARHSP1, ↓CISD1, ↓CTSF, ↑DHDH, ↑DHRS1, DHRS9, ↓ENSA, FXYD3, ↓HHATL, ↓HIBADH, ↑IAH1, ↑ITIH5, ↓LRPPRC, MATN4, MCM2, ↑PMM1, POU4F2, ↑SCPEP1, ↑SERPINH1, ↓SNAP91, ↓SYT1, SYT6, TP53, ↓TRIM46, TSC22D1	20	18	Cell Morphology, Cellular Function and Maintenance, Auditory and Vestibular System Development and Function
☐	25	↓ACO2, Aconitase, ↑APOH, ↑BGN, ↑C3, ↑C4A/C4B, C4BP, ↑COL14A1, Collagen type II, Collagen type III, Collagen type ix, Collagen(s), Cpla2, creatine kinase, EF-1 alpha, Fc gamma receptor, ↑GC, GPIIB-IIIA, HDL-cholesterol, ↑HDLBP, ↑HSPG2, Kallikrein, ↑LUM, ↑Mcpt4, ↓MYEF2, ↑OGN, P38 MAPK, ↑PRELP, ↑PSAT1, SAA, Serine Protease, ↑SERPINC1, SYK/ZAP, Tcf 1/3/4, ↑TTR	18	17	Hair and Skin Development and Function, Connective Tissue Disorders, Organ Morphology

©2000-2017 QIAGEN. All rights reserved.
