# Supplementary material for: Region-Resolved Quantitative Proteome Profiling Reveals Molecular Dynamics Associated With Chronic Pain in the PNS and Spinal Cord
Source: Front Mol Neurosci. 2018 Aug 14;11:259. doi: 10.3389/fnmol.2018.00259 (PMC6103001; doi:10.3389/fnmol.2018.00259)
Supplement: Supplementary Table 12 — Top biological networks harboring SN proteins significantly regulated upon SNI. [file Table_12.pdf]

Top biological networks harboring differentially regulated proteins in SN

Network List

IPA Build version: 460209M

ID	Molecules in Network	Score	Focus Molecules	Top Diseases and Functions
1	↑ANXA1, ↓C1orf123, ↑CORO7/CORO7-PAM16, ↑DDX39B, ↑DDX3X, ↑ENTPD2, ↑FLNA, ↑GAS7, ↓GYG1, H2AF, ↑H2AFY, ↑H2AFZ, ↑HNRNPL, ↑IQGAP1, ↑KHDRBS1, ↑LMNA, ↑MFAP5, ↑MYH9, ↓MYH10, ↓MYO1C, ↑NONO, nucleoside-triphosphatase, ↓OXR1, ↓PLEKHB1, ↑PPP1CA, ↑RBM14, ↑RUVBL1, ↑S100A4, ↑SEC13, Sfk, ↑SFPQ, ↓SORD, ↑SRSF1, ↑TGM2, ↑TWF1	43	32	Cellular Movement, Cellular Assembly and Organization, RNA Post-Transcriptional Modification
2	Abl1/2, AChR, ↓ADSS, ↓ASPN, ↓CADM1, ↓CADPS2, ↑CRIP1, ↓CTNNB1, ↓CYP51A1, ↓DAG1, ↑DMD, ↓DRP2, ↑GATM, Lamin, ↑LAP3, ↑LMNB1, ↑LMNB2, ↓MAP4, ↓MPP6, ↑NDRG2, ↑PRKAR1A, ↑PRKCSH, ↓PRX, ↑RBM39, ↑RCN1, ↑SCARB2, SerineProtease, ↓SGCD, ↓SNTA1, ↓SNTB1, ↑STARD10, ↓SYNM, ↑THOP1, ↑TMPO, ↓UQCRB	41	31	Organ Morphology, Skeletal and Muscular System Development and Function, Cell Morphology
3	60Sribosomal subunit, ↓CHAT, ↓ENO2, MAP3K, MIRLET7, Ras, ↑RPL3, ↑RPL5, ↑RPL7, ↑RPL9, ↑RPL11, ↑RPL12, ↑RPL14, ↑RPL15, ↑RPL17, ↑RPL18, ↑RPL19, ↑RPL21, ↑RPL22, ↑RPL24, ↑RPL26, ↑RPL27, ↑RPL28, ↑RPL30, ↑RPL31, ↑RPL10A, ↑RPL13A, ↑RPL18A, ↑RPL27A, ↑RPL35A, ↑RPL37A, ↑RPL7A, ↑RPLP2, ↓RTCA, transglutaminase	38	30	Cancer, Cell Death and Survival, Organismal Injury and Abnormalities
4	↓AARS, ↓CAND1, ↑EEF1A1, ↑EEF1B2, ↑EEF1D, ↑EEF1G, ↑EPRS, ↑GPX7, hnRNP H, ↑HNRNPH1, ↑HNRNPUL2, IKK (complex), ↑KHSRP, ↑LARS, ↓MYO5A, P glycoprotein, ↓PDCD5, ↑RARS, ↑RNASE4, RNase A, ↑RPS9, ↑RPS14, ↑RPS16, ↑RPS25, ↓RTN1, ↓SDCBP, ↑SUMO2, ↑TARDBP, ↓TOM1L2, ↑TOP2B, ↑UBA2, ↓UBE2N, ↓UBE2V1, Ubiquitin, ↑VARS	38	30	Cellular Assembly and Organization, Molecular Transport, Nucleic Acid Metabolism
5	6-phosphofructokinase, ↓AAMDC, ↓ASL, ↓ATIC, ↑BZW2, Cytoplasmic Dynein, ↓DMXL2, ↓DYNC1H1, ↓DYNC1LI1, ↓DYNLRB1, ↓DYNLT3, ↑EGFR, ↓EPB41, Flotillin, ↓FN3KRP, ↑ITPA, ↑MATN2, ↑MATR3, ↓NCDN, ↓NTPCR, ↓PAICS, ↑PFAS, PFK, ↓PFKL, ↓PFKM, ↓PFKP, phosphofructokinase, ↓RAB18, ↑RAB22A, ↓RAB3GAP1, ↓SLC25A32, ↓SLC4A1, tubulin (family), ↓UROD, ↑VPS25	36	29	Carbohydrate Metabolism, Small Molecule Biochemistry, Developmental Disorder
6	7S NGF, ↓ANXA11, AP-3, ↑AP3B1, ↑ATXN2L, Beta Tubulin, ↓CAV2, ↓CDC42EP4, ↑CELF2, ↑CORO1B, CRMP, ↑CRMP1, ↓DPYSL2, ↓DPYSL3, ↓EML1, ↓EPS15L1, ↓HEBP2, ↓INA, Jnk, ↓LRSAM1, ↑MICAL1, ↓NEFH, ↓NEFL, ↓Nefm, ↓PDCD6, ↓PRKCDBP, ↓PRPH, ↓PTRF, ↓SDPR, ↑SEC31A, ↑SEPHS1, ↑SEPT9, TEAD, ↓TUBB3, ↓TUBB2B	36	29	Cellular Assembly and Organization, Cellular Function and Maintenance, Nervous System Development and Function
7	Ampa Receptor, ↑Anp32b, ↓COX5A, ↓COX6A1, ↓COX6B1, ↓GNA11, ↑GOLPH3, Gαq, ↑HDLBP, ↑HEXA, ↑HIST1H2BO, ↑HIST2H2BF, ↑Hnrnpa1, ↑Hnrnpa3, ↑HNRNPAB, ↑HNRNPD, ↑HP1BP3, ↑IPO4, mGluR, Plc beta, ↓PLCB1, ↑PTBP1, ↑SAFB, ↑Sf1, SRC (family), ↑SRSF2, ↑SRSF6, ↑SRSF7, ↑SYNCRIP, ↑THRAP3, TRAP/Media, ↑U2AF2, ↑VPS35, ↑VPS26A, ↓WASHC5	36	29	RNA Post-Transcriptional Modification, Molecular Transport, RNA Trafficking
8	↓ABAT, ↓ADAM10, ↓ADAM22, ↓ADAM23, ↓ATP2B2, ↑BASP1, ↓BCAM, ↑CACNA2D1, ↓CALB2, Calmodulin, ↓CD9, ↑CD44, ↓CD81, ↑CNDP2, Collagen type IV, ↑DCLK1, ↑FAM98B, ↑HIST1H2BB, ↓ISLR, ↓ITGA6, ↓ITGB4, Iti, ↑ITIH4, ↑ITIH5, ↑LAMA5, Laminin, Laminin1, ↓LGI3, ↑LMAN2, Metalloprotease, ↓MYO1B, ↓MYO1D, ↑PLG, ↑SGPL1, ↓TSPAN15	36	29	Cell-To-Cell Signaling and Interaction, Tissue Development, Cell Morphology
9	calpain, ↓CAPN2, Cathepsin, ↑CTSB, ↑CTSC, ↑CTSD, ↑CTSZ, ↑DHX15, ↑EEF2, ↑EFTUD2, ↑Eif1, ↑EIF4A3, ENaC, ↑ETF1, ↓GLRX3, ↑GSPT1, ↓HBA1/HBA2, HLA-DR, ↑HNRNPC, ↑HYOU1, ↑LSM2, ↑LSM4, p85 (pik3r), ↑PABPC1, ↑PCBP1, ↑PRPF19, snRNP, ↑SNRPB, ↑SNRPD2, ↑SNRPD3, ↑SNRPF, ↑SNRPN, ↑TPP1, ↓UGP2, ↑UPF1	36	29	RNA Post-Transcriptional Modification, Cell Cycle, DNA Replication, Recombination, and Repair
10	↓ALDOC, ↑APEX1, ↓ATG3, ↓ATP1A1, ↑CA3, Calcineurin B, Cbp/p300, ↓CBR1, ↓DBI, ↑DHX9, ↓DUSP15, EGLN, ↓FASN, ↓GABARAPL2, ↑GANAB, ↑HNRNPA2B1, ↓IDI1, ↑INPP5F, ↓MDP1, ↓MPI, ↓MTMR2, MTORC1, ↓NIPSNAP1, p70S6k, ↓PGM1, phosphatase, ↓PPP3CA, ↑RPN1, ↓SCCPDH, ↑SET, ↑SF3A1, ↓SORBS3, ↓SYNJ1, ↑TCEA1, ↓TNS3	36	29	Carbohydrate Metabolism, Lipid Metabolism, Small Molecule Biochemistry
11	14-3-3, alcohol group acceptor phosphotransferase, Alpha tubulin, ↑ARL2, ↑CAPZB, ↓CNP, ↑DAD1, Dynein, ↓EML2, Gamma tubulin, ↑HPRT1, ↓MAP1B, ↓MAPK3, ↑MTHFD1L, ↓PAFAH1B1, ↓PEX14, ↑PFN1, ↑RBM3, ↑SEC61A1, ↑SEC61B, ↓SIRT2, somatostatin receptor, ↑SSR1, ↓SYNE2, ↑TCP1, ↓TPPP, ↓TUBA8, ↓TUBA4A, ↓TUBB, ↓TUBB1, ↓TUBB6, ↓TUBB2A, ↓TUBB4A, ↓TUBB4B, tubulin	34	28	Cancer, Hematological Disease, Immunological Disease
12	↑AEBP1, ↓CNTFR, ↓Cops2, ↓COPS3, ↓COPS4, Cytochrome bc1, cytochrome-c oxidase, ↑DDOST, dolichyl-diphosphooligosaccharide-protein glycotransferase, E3 RING, ↑ESYT1, ↓ETFA, ↑GPC1, ↓GPS1, ↑HIST1H2BN, ↓ITM2C, ↑Ktn1, ↓MAOA, Mapk, Mitochondrial complex 1, NADH dehydrogenase, ↓NDUFA12, ↓NDUFAB1, ↓NDUFB8, ↓NDUFS1, ↓OSBPL1A, Plc delta, ↑RPN2, ↓SKP1, ↓STOML2, ↑STT3A, ↑TMED2, ↑TMEM43, ↓VAPB, ↑YIF1B	32	27	Post-Translational Modification, Developmental Disorder, Hereditary Disorder
13	↑BANF1, ↓CAMK2G, ↑CBX3, ↑CBX5, ↑CFB, ↑DDX5, ↑DDX17, ↑DEK, ↑EMD, ↑FBL, ↑Fus, ↑HDGF, ↓HIST2H2AC, histone deacetylase, Histone h4, ↑HNRNPM, HP1, ↑HPX, ↑ILF3, ↑MAGOH, NuRD, ↓PLS3, ↑RBBP4, ↑RCC2, ↑RPL10, ↑SAMHD1, ↑STAT1, ↑SUB1, TFIIH, TH2 Cytokine, thymidine kinase, Top2, ↑TRIM28, ↓UBE4A, ↓UFD1L	32	27	RNA Post-Transcriptional Modification, Cancer, Gastrointestinal Disease
14	↑ANO6, ↓ATXN10, ↓BSG, ↓CDK5, ↓DPP8, ↑EIF3E, ↑ELAVL1, ↓FAM114A2, ↑GIT1, ↓GLOD4, ↓HIBADH, Histone h3, ↑HNRNPU, ↑HSPB1, Interferon alpha, ↓ISOC1, ↓KRAS, ↑LRP1, ↑LTA4H, ↓MCTS1, ↑NEDD4L, NFkB (family), Notch, ↓PIGN, Pro-inflammatory Cytokine, RNA polymerase II, ↑RPL13, ↑SERPINA1*, ↑SF3B1, ↓TBCB, TCR, Tlr, ↓UBL3, ↑VTN, ↓ZC2HC1A	32	27	Neurological Disease, Skeletal and Muscular Disorders, Endocrine System Disorders
15	↓ACO2, Aconitase, Alpha catenin, ↓ANK1, ↓ANK3, Ap2alpha, ↓ATP1A2, ↓ATP1A3, ↓ATP1B1, ↓ATP1B3, AURK, ↓CADM4, ↑CORO1C, ↓EPB41L2, ↓EPB41L3, ↑FLOT2, ↓FSTL1, ↓IGFBP6, ITPR, ↓KIF5C, Mapre, ↓Mapre2, ↓MAPRE3, ↑MYEF2, ↑MYO18A, Na-k-atpase, ↓NEXN, P38 MAPK, ↓PIP4K2A, ↑SORBS2, Spectrin, ↓SPTA1, ↓SPTAN1, ↓SPTB, ↓SPTBN1	30	26	Cellular Function and Maintenance, Small Molecule Biochemistry, Molecular Transport
16	↑Alyref, Angiotensin II receptor type 1, ↓APOO, ↑ARCN1, Arf, ↓C19orf70, ↑CALR, ↓COL15A1, ↑COL1A2, ↑COL4A2, ↑COL6A1, Collagen Alpha1, COP I, ↑COPA, ↑COPB1, ↑DBNL, ↑EFEMP1, ↓ENDOD1, Focal adhesion kinase, ↑GORASP2, ↓KIF21A, ↓KRT73, Lpa receptor, MHC Class I (complex), PDGF (family), ↑PDIA3, ↑PDIA4, ↑PURB, ↑SARS, ↑SEC23B, ↑SERPINH1, Tap, ↑THBS4, ↑TMED10, ↑USO1	30	26	Cancer, Connective Tissue Disorders, Organismal Injury and Abnormalities
17	↓AAK1, ↓AHSA1, ↓AVIL, ↓BAG3, Beta Arrestin, ↓CHORDC1, Clathrin, ↓DNAJB4, ↓DNAJC6, ↓DNAJC7, ↓ECHDC1, ↓FKBP4, Gpcr, Hdac, HSP, Hsp70, Hsp90, Hsp22/Hsp40/Hsp90, ↓HSP90AB1, ↑HSP90B1, ↓HSPA8, ↑HSPA9, ↓HSPA12A, ↓HSPA4L, ↓HSPB8, ↓HSPH1, ↓LIMCH1, MHC Class II (complex), ↑NACA, ↓NAP1L1, ↑PPP5C, ↑SAE1, ↓ST13, ↓STIP1, ↓SUGT1	30	26	Post-Translational Modification, Protein Folding, Drug Metabolism
18	19S proteasome, 20s proteasome, adenosylhomocysteinase, ↑ADRM1, ↓AHCY, ↓AHCYL1, ↓AHCYL2, ATPase, ↑CAPG, ↑CROCC, Cytokeratin, Foxo, ↓KIAA0368, ↑MCAM, ↓OLA1, ↓OSTF1, PI3K (complex), PSMA, PSMC, ↑PSMC2, ↑PSMC3, ↑PSMC5, ↑PSMC6, PSMD, ↑PSMD3, ↓PSMD5, ↑PSMD6, ↑PSMD7, ↓PSMD9, ↑PSMD11, ↑PSMD13, ↑PSME2, ↓UBE2O, ↓UBQLN1, ↓USP14	28	25	Cancer, Cell Death and Survival, Organismal Injury and Abnormalities
19	amylase, ↑Anp32a, ↓AP1B1, ↓AP2A1, Beta adaptin, C4BP, ↓CADPS, Collagen type ix, Collagen type V, ↓CPLX1, ↑ESD, ↑FARSB, ↑FN1, GABA-A receptor, ↑H2AFV, ↑HIST1H2BK, ↓KRT81, MIR200, ↓MTAP, ↑Mug1/Mug2, ↓NAPB, ↓NAPG, ↓NSF, phosphorylase, ↓PYGB, ↓PYGM, Rab5, ↓RAB3A, ↓SLC5A3, ↓SNAP25, Snare, ↓STXBP1, ↓SYN2, ↑TPD52L2, ↓YKT6	28	25	Cellular Assembly and Organization, Cellular Function and Maintenance, Molecular Transport
20	Caveolin, ↓CD2AP, Cr3, ↓DLST, ↓DNM1, ↓DNM2, ↓DNM3, Dynamin, ↓EHD1, ↓EHD2, ↓EHD3, ↓EHD4, Endophilin, ERK1/2, ↑GLIPR2, growth factor receptor, ↓JAM3, ↑LRRC59, ↓MICAL3, ↓MYO1E, ↓NQO2, ↓PACSIN1, ↓PACSIN2, ↓PACSIN3, ↓PCSK1N, ↓PFN2, PKC alpha/beta, Pki, ↓PRPSAP1, ↓PRPSAP2, ↓SH3GL2, ↓SH3GLB2, SUMO, Syntaxin, Vla-4	27	24	Cellular Function and Maintenance, Cellular Assembly and Organization, Nervous System Development and Function
21	↓AACS, ↓ACSL1, ↓ACSL4, adenosine-tetraphosphatase ↑APOA4, ↑APOD, ATP synthase, ↓ATP5H, ↓ATP5J, ↓ATP5O, ↑C1QBP, ↓C21orf33/LOC102724023, ↑CP, ↓DLAT, ↓EPHX1, ↑ERH, F1 ATPase, ↑FABP3, Ferritin, ↑IDH1, Insulin, MIR124, N-cor, NCOR-LXR-Oxysterol-RXR-9 cis RA, Nr1h, ↑Orm1 (includes others), ↓PDHB, Pdi, ↑PGD, ↑PGK1, ↓PHYHIPL, ↑SCAMP2, Srebp, ↓STRAP, ↓VSNL1	27	24	Lipid Metabolism, Small Molecule Biochemistry, Molecular Transport
22	↓AKR1B1, ↑ANXA5, ↓BCAT1, Caspase 3/7, ↑CBWD1, ↓CLIP2, ↑CSNK1A1, ↑CYP2J2, ↑EIF2S1, Fcer1, ↓FGF1, ↓GCLC, ↓GCLM, Gm-csf, ↓GPC6, ↑HNRNPK, Ikb, LDL, ↓MAP2K2, ↓MAP2K4, MAP2K1/2, Mek, ↑MRC1, ↑MVP, ↑NCL, PI3K (family), ↑PREP, ↓PRKCD, Proinsulin, ↑RPL23, ↑RPS3, ↑RPS6, Rsk, Shc, ↓TTC9	27	24	Protein Synthesis, Cardiovascular Disease, Organismal Injury and Abnormalities
23	aldehyde dehydrogenase, aldehyde dehydrogenase (NAD), ALDH, ↓ALDH2, ↓ALDH1A1, ↓ALDH1L1, ↓ALDH6A1, ↓ALDH7A1, ↓ALDH9A1, ↑API5, C3-Cfb, ↑CFH, ↑CLIC1, ERK, ↓ETFB, ↓FH, glutathione transferase, GOT, ↓GOT1, ↓GOT2, ↓Gsta4, ↓GSTM3, ↑INPP1, IQGAP, ↑IQGAP2, malate dehydrogenase, ↓MDH1, ↓MDH2, ↓PC, ↓PCP4, ↓PITPNA, ↓PLXNB1, RhoGap, S100, TMSB4	25	23	Small Molecule Biochemistry, Cellular Function and Maintenance, Energy Production
24	↓ABLIM2, ↑ACTR2, ↑ACTR3, Alpha 1 antitrypsin, Alpha actin, Arp2/3, ↑ARPC2, ↑ARPC3, ↑ARPC4, ↓ARPC1A, ↑ARPC1B, ↑COTL1, Ctnna, ↓CTNNA2, ↓CTNNA3, ↑DBN1, ↑F2, F Actin, G-Actin, GST, Myosin2, ↓NCKIPSD, NWASP, ↑PDLIM1, ↓PGM2L1, phosphoinositide phospholipase C ↓PLCB3, ↑PLCD1, Pmca, Profilin, ↑RNH1, ↑SERPING1, ↑SRM, ↑TAGLN2, ↓TAGLN3	25	23	Cellular Assembly and Organization, Cellular Function and Maintenance, Tissue Development
25	↑ABCA6, ↑ACADS, ↑APOA1, ↑APOE, ↑C3, ↓CALCB*, chemokine, ↓Crip2, ↑DPEP1, ↓ECI1, ↑FAM129A, ↑FTL, ↓GPX4, HDL, HDL-cholesterol, hemoglobin, ↑HP, IL-2R, IL12 (complex), ↑LGMN, ↑MAP7D1, mediator, ↑MVK, Nos, ↓PHGDH, ↑PRDX1, ↓PRDX5, ↓RIDA, SAA, ↓SKIV2L2, ↑STEAP3, T3-TR-RXR, Tnf receptor, ↑TXN, VLDL	25	23	Free Radical Scavenging, Small Molecule Biochemistry, Connective Tissue Development and Function

©2000-2017 QIAGEN. All rights reserved.
