# Supplementary material for: Region-Resolved Quantitative Proteome Profiling Reveals Molecular Dynamics Associated With Chronic Pain in the PNS and Spinal Cord
Source: Front Mol Neurosci. 2018 Aug 14;11:259. doi: 10.3389/fnmol.2018.00259 (PMC6103001; doi:10.3389/fnmol.2018.00259)
Supplement: Supplementary Table 13 — Top biological networks harboring SC proteins significantly regulated upon SNI. [file Table_13.pdf]

Top biological networks harboring differentially regulated proteins in SC

Network List

IPA Build version: 460209M

☐	ID	Molecules in Network	Score	Focus Molecul	Top Diseases and Functions
☐	1	26sProteasome, Actin, Akt, Alpha catenin, ↑ANLN, ↓BGN, calpain, caspase, ↓CKM, ↑CNTN1, ↑CNTNAP1, ↓COL6A1, ↓COL6A2, Collagen type VI, ↓ENO1, ↑EPRS, F Actin, ↓GAPDH, ↓Hbb-b1, Hsp70, ↓HSP90AB1, Immunoglobulin, ↓LUM, ↑MYH10, ↑MYH14, ↑MYO1D, ↑MYO5A, ↑OGDH, ↓PRPH, ↓PRX, RNA polymerase II, ↑SPTBN1, ↓Tmsb4x(includesothers)*, ↓Tpm1, Ubiquitin	57	23	Connective Tissue Disorders, Immunological Disease, Organismal Injury and Abnormalities
☐	2	20sproteasome, ↑ACO2, Alpha tubulin, ↓BAG3, Beta Tubulin, Calcineurin protein(s), Ck2, cytochrome C, Cytoplasmic Dynein, ↑DCTN1, ↑DMXL2, ↑DYNC1H1, Dynein, ERK1/2, ↓FKBP1A, Hsp90, ↓MAP4, ↓MAPT, mediator, ↑MFN2, Myosin, ↓NOL3, ↓Pcp4l1, ↑PLEC, ↓PPIA, ↓PVALB, ↓SNCB, ↓SNCG, ↓STMN1, ↓TUBB6, ↓TUBB2A, ↓TUBB2B, ↓TUBB4A, tubulin, tubulin (family)	50	21	Neurological Disease, Organismal Injury and Abnormalities, Psychological Disorders
☐	3	AMPK, ANXA9, benzylamine, CD3, CDC42SE1, ↓COX5A, ERK, estrogenreceptor, Fe3+, ↓FGF1, FGFBP1, gelatinase, ↓GFER, GJC1, Insulin, Jnk, ↓KSR2, ↑MAOB, Mapk, Mek, NFkB (complex), NTF3, P38 MAPK, Pdgf Ab, phosphocreatine, PI3K (complex), Pka, Ras, RGS2, Sos, ↓STMN1, THPO, tyramine, Vegf, VEGFD	6	4	Energy Production, Molecular Transport, Cell Cycle
☐	4	↓ANXA11, ↓ARF4, ↑BCAN, ↑C1QA, CCNYL1, CHRNA4, cyanocobalamin, ↓EGFR, Endothelin, FBLN2, FRAT2, ↓GCLM, ↓GFER, ↑HAPLN4, IGKC, IL4I1, L-dopa, ↓LAMA4, ↓LGMN, linolenic acid, magnesium, MDK, NAGLU, ↑NCLN, ↑NOMO1(includesothers), ↓PMP2, stearic acid, TNF, ↑TNR, ↓TNS3, TOM1L1, TRPC3, TRPC6, ↓VAT1L, ZNRF1	6	4	Nervous System Development and Function, Organ Morphology, Organismal Development

©2000-2017 QIAGEN. All rights reserved.
